# SET8 inhibition preserves PTEN to attenuate kidney cell apoptosis in cisplatin nephrotoxicity

**DOI:** 10.1038/s41419-025-07526-y

**Published:** 2025-03-31

**Authors:** Xu Yang, Yingjie Guan, George Bayliss, Ting C. Zhao, Shougang Zhuang

**Affiliations:** 1 Department of Medicine, Rhode Island Hospital and Alpert Medical School, Brown University, Providence, RI USA; 2 Department of Plastic Surgery, Warren Alpert Medical School, Brown University, Providence, RI USA; 3 Department of Nephrology, Shanghai East Hospital, Tongji University School of Medicine, Shanghai, China

**Keywords:** Acute kidney injury, Methylation

## Abstract

The aberrant expression of SET8, a histone methyltransferase that mediates H4 lysine 20 mono-methylation (H4K20me1), is implicated in the pathogenesis of various tumors, however, its role in acute kidney injury (AKI) is unknown. Here, we showed that SET8 and H4K20me1 were upregulated in the murine kidney with AKI induced by cisplatin, along with increased renal tubular cell injury and apoptosis and decreased expression of E-cadherin and Phosphatase and Tensin Homolog (PTEN). Suppression of SET8 by UNC0379 improved renal function, attenuated tubule damage, and restored expression of PTEN but not E-cadherin. UNC0379 was also effective in lessening cisplatin-induced DNA damage response (DDR) as indicated by reduced expression of γ-H2AX, p53, p21, and alleviating cisplatin-impaired autophagy as shown by retained expression of Atg5, Beclin-1, and CHMP2A and enhanced levels of LC3-II in the kidney. Consistently, inhibition of SET8 with either UNC0379 or siRNA mitigated apoptosis and DDR and restored autophagy, along with PTEN preservation in cultured renal proximal tubular epithelial cells (TKPTs) exposed to cisplatin. Further studies showed that inhibition of PTEN with Bpv or siRNA potentiated cisplatin-induced apoptosis and DDR, hindered autophagy, and conversely, alleviated by overexpression of PTEN in TKPTs. Finally, blocking PTEN largely abolished the inhibitory effect of UNC0379 on apoptosis. Taken together, these results suggest that SET8 inhibition protects against cisplatin-induced AKI and renal cell apoptosis through a mechanism associated with the preservation of PTEN, which in turn inhibits DDR and restores autophagy.

## Introduction

Cisplatin is one of the most widely used chemotherapy drugs to treat various tumors [1]. However, the uptake of cisplatin by renal proximal tubules results in cellular death and acute kidney injury (AKI) [2]. AKI affects ~10–20% of cancer patients who receive cisplatin treatment [3]. To date, the pathogenesis of cisplatin-induced AKI remains incompletely understood, and effective renoprotective approaches are not available.

Accumulation of cisplatin in renal tubular cells induces various intracellular stress responses, including DNA damage response (DDR) and autophagy [4]. DDR is a cellular event leading to DNA repair, cell cycle arrest, senescence, and/or apoptosis, which is orchestrated by various signaling cascades, including phosphorylation of the histone variant H2A (γ-H2AX) and p53 [5]. γ-H2AX is an early step in the cellular response to DNA damage and is involved in the initiation of DDR, while phosphorylation of p53 is a critical downstream effector of DDR and is responsible for triggering cell cycle arrest by upregulation of p21 and inducing apoptosis by activation of caspase 3 [5, 6]. In cisplatin nephrotoxicity, autophagy is also rapidly activated in kidney tubular cells and is required to protect the kidney from injury and apoptosis [7]. However, the precise mechanisms that underlie DDR and autophagy induction by cisplatin in renal epithelial cells are still elusive.

Recent studies have suggested the importance of phosphatase and tensin homolog (PTEN) in regulating kidney cell apoptosis via p53 and autophagy in AKI. It has been reported that pharmacological inhibition of PTEN aggravates cisplatin-induced AKI and tubular cell apoptosis by activation of p53 signaling pathways [8], while activation of PTEN protects the kidney against AKI apoptosis by promoting autophagy [9]. Moreover, PTEN overexpression promotes renal epithelium repair by restoring CHMP2A-mediated phagosome closure [10]. Although PTEN expression and activation are indispensable for renoprotection, it is often downregulated in the kidney with AKI-induced diverse insults, including cisplatin [8, 10, 11]. Thus, identifying and targeting the mechanisms that lead to the loss of PTEN in the kidney may be a novel strategy to treat AKI.

PTEN stability and enzymatic activity are regulated by multiple mechanisms, including epigenetic silencing and post-transcriptional and posttranslational modifications [12]. Among several types of posttranslational modifications, histone methylation has been shown to regulate cells’ sensitivity to DNA-damaging agents, including chemotherapeutics and radiotherapeutics. For example, NSD2, a methyltransferase, can induce PTEN methylation and subsequently recruit PTEN to DNA damage sites to regulate DDR and cellular sensitivity to DNA damaging agents in tumor cells [13]. SET8 (also known as PR-set7/9, SETD8, KMT5A), another member of the SET8 domain-containing methyltransferase family, specifically catalyzes mono-methylation of histone H4 lysine 20 (H4K20me1) [14] and has been implicated in multiple biological processes, including DNA replication, DNA damage repair, and gene transcription [15–17]. However, the role of SET8 in AKI and renal epithelial cell death has not been investigated yet.

In this study, we explored the role and mechanism of SET8 in cisplatin nephrotoxicity. Our results showed that cisplatin treatment increased expression of SET8 and H4K20me1 with downregulation of PTEN in the kidneys of mice and cultured murine proximal tubular epithelial cells (TKPTs). Targeted inhibition of SET8 attenuates AKI and reduces renal tubular cell apoptosis via a mechanism associated with PTEN preservation and subsequent suppressing DNA damage and retaining autophagy.

## Results

### Inhibition of SET8 by UNC0379 attenuates cisplatin-induced AKI in mice

**Fig. 1:**
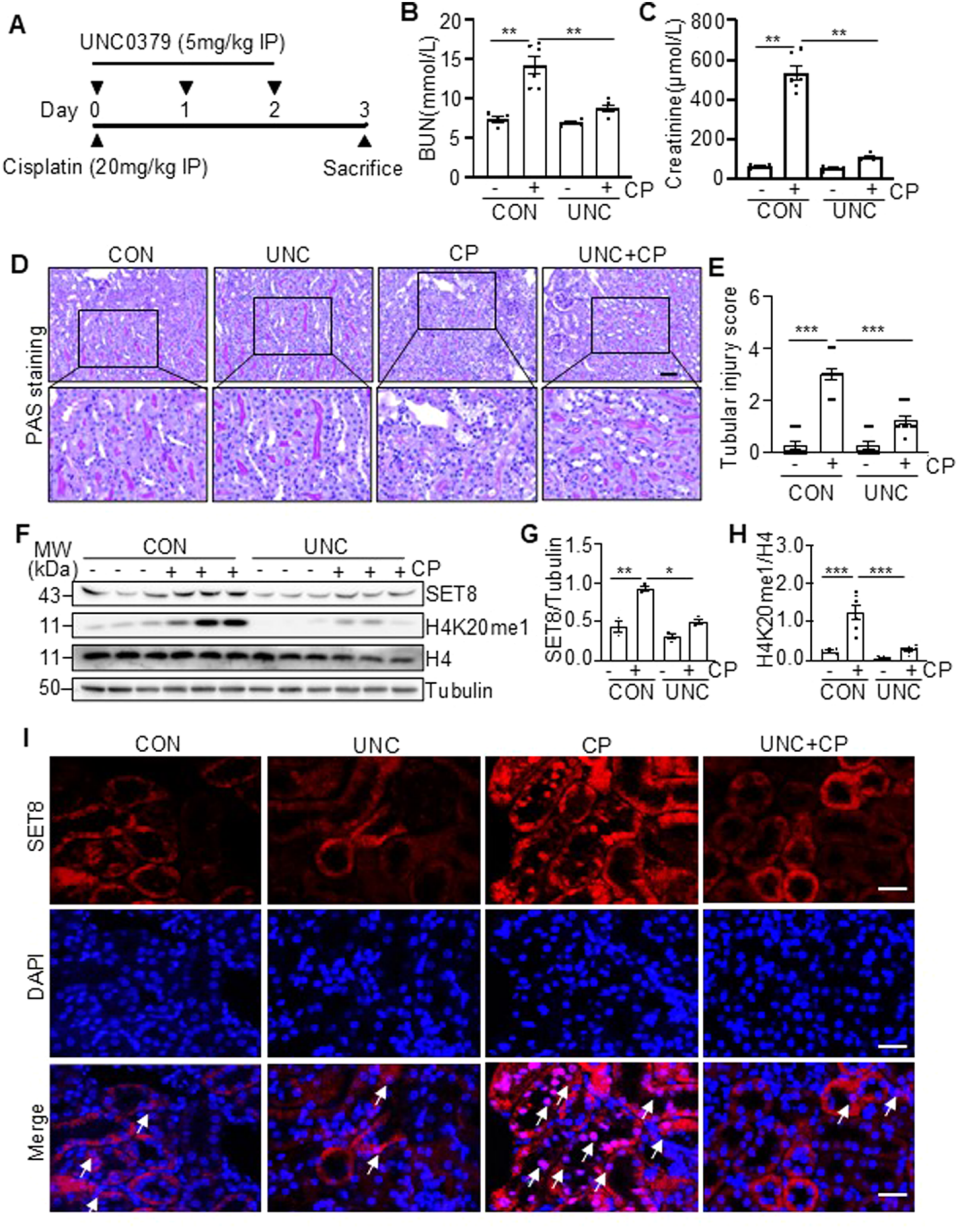
Administration of UNC0379 (UNC) improves renal function and attenuates renal pathology in cisplatin (CP)-induced AKI in mice. **A** The diagram depicts the treatment scheme with CP and UNC0379. On day 3 after CP treatment, blood samples and kidney tissues were collected for further analysis. **B**, **C** Blood urea nitrogen (BUN) and serum creatinine (SCr) were detected as measures of kidney function (*n* = 6 for each group). **D** Representative sections of Periodic acid-Schiff (PAS) staining of kidney tissues (magnification ×200, ×400, respectively). Scale bars = 50 μm. **E** The degree of tubular injury was scored using the method described in the “Materials and Methods” section (*n* = 6 for each group). **F** Kidney tissue lysates from four groups were subjected to immunoblot analysis using antibodies against SET8, H4K20me1, H4, and Tubulin. The levels of SET8 (**G**) and H4K20me1 (**H**) were quantified by densitometry and normalized with Tubulin and H4, respectively. **I** Immunofluorescent (IF) staining for SET8. DAPI staining was used to localize nuclei in TKPTs. Scar bars = 20 μm. Arrows: nuclei with SET8 staining. Data are represented as the mean ± SEM of at least three experiments. *P < 0.05, **P < 0.01, ***P < 0.001. IP intraperitoneal injection.

To determine the role of SET8 in AKI, we examined the effect of SET8 inhibition on cisplatin-induced AKI in a mouse model using UNC0379, a highly selective inhibitor of SET8 [18–20]. As depicted in Fig. 1A, UNC0379 was administered once daily for three consecutive days. Blood urea nitrogen (BUN) and serum creatinine (SCr) were measured to reflect the changes in renal function. The results in Fig. 1B, C demonstrated a significant elevation in BUN and SCr levels in the cisplatin group compared to the control group, which is consistent with progressive renal tubular injury as indicated by tubular distention, swelling and necrosis, and loss of brush border (Fig. 1D, E). Administration of UNC0379 significantly reduced the levels of BUN and SCr and attenuated renal tubular damage in mice following cisplatin treatment (Fig. 1B–E).

Western blot analysis of whole kidney lysates revealed that cisplatin treatment resulted in increased expression of SET8 and its epigenetic activation marker, H4K20me1, in the injured kidney, compared with sham controls. Administration of UNC0379 reduced cisplatin-induced upregulation of SET8 and H4K20me1 without alteration of histone H4 levels (Fig. 1F–H). Immunofluorescence (IF) staining indicated that SET8 was predominantly expressed in renal tubular cells, with most being distributed in the cytoplasm and a small portion in the nucleus in vehicle or UNC0379-treated kidneys. However, SET8 was largely observed in the nucleus of renal tubular cells following cisplatin treatment, and UNC0379 inhibited this response (Fig. 1I).

Collectively, these data indicate that inhibition of SET8 with UNC0379 improves renal function and mitigates renal tubule damage in mice with cisplatin treatment, suggesting that SET8 activation contributes to the development of AKI.

### UNC0379 inhibits renal tubular cell injury and apoptosis and preserves PTEN expression in the murine kidney following cisplatin treatment

**Fig. 2:**
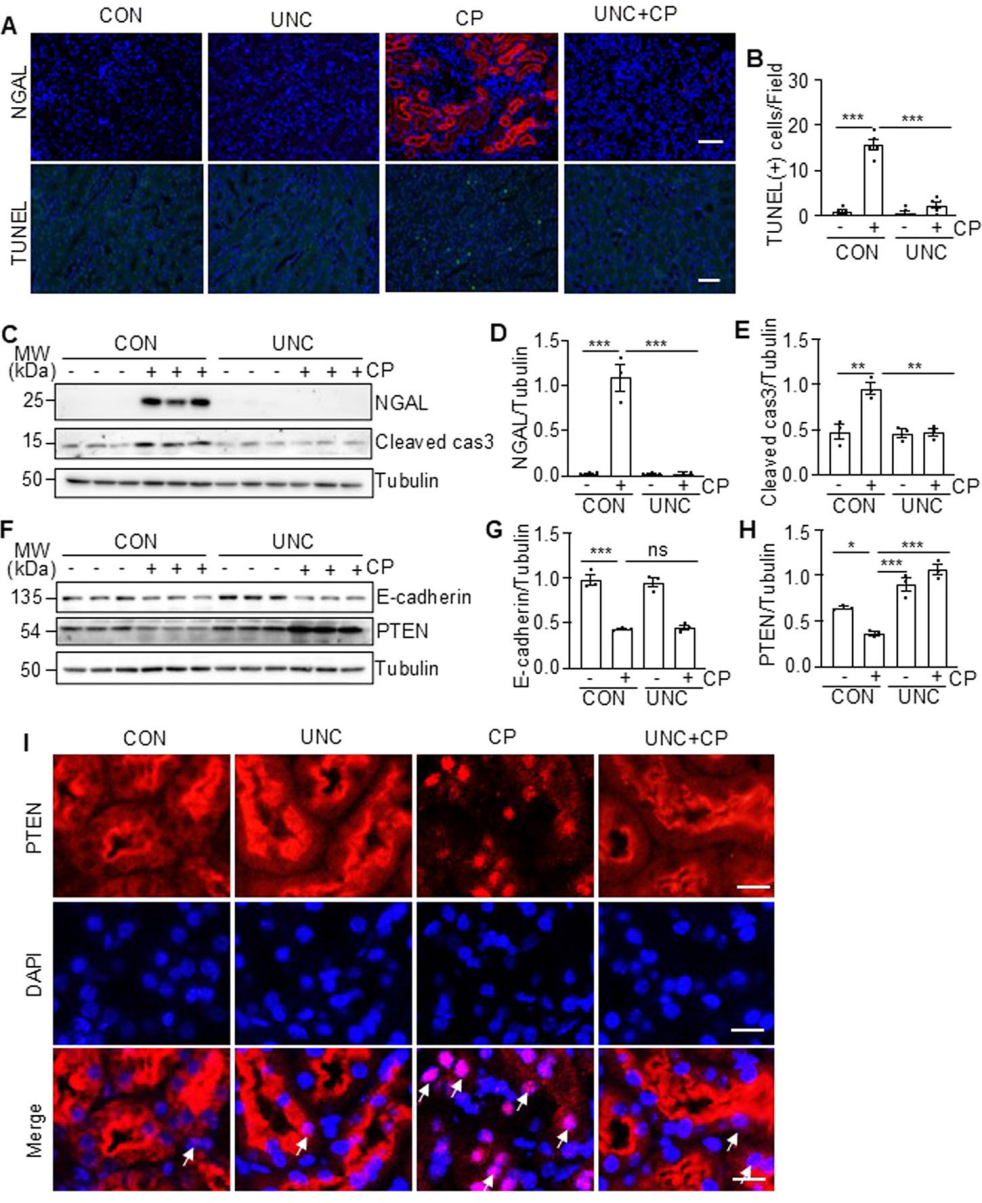
Pharmacological inhibition of SET8 attenuated cisplatin (CP)-induced renal tubular cell apoptosis in mice. **A** Kidney tissue was collected and subjected to neutrophil gelatinase-associated lipocalin (NGAL) and TdT-mediated dUTP nick-end labeling (TUNEL) staining. Scar bars = 50μm. **B** The number of TUNEL-positive cells per field was counted in at least 10 fields per section. DAPI staining was used to localize nuclei in TKPT. Scale bars = 50 μm. Kidney tissue lysates were subjected to immunoblot analysis using antibodies against NGAL, cleaved caspase3 (Cleaved cas3), and Tubulin (**C**). The levels of NGAL (**D**) and Cleaved cas3 (**E**) were quantified by densitometry and normalized with Tubulin. Kidney tissue lysates from four groups were subjected to immunoblot analysis using antibodies against E-cadherin, Phosphatase and Tensin Homolog (PTEN), and Tubulin (**F**). The levels of E-cadherin (**G**) and PTEN (**H**) were quantified by densitometry and normalized with Tubulin. **I** Immunofluorescent (IF) staining for PETN. DAPI staining was used to localize nuclei in TKPT. Scale bars = 20 μm. Arrows: Nuclei with PETN staining. Data are represented as the mean ± SEM of at least three experiments. *P < 0.05, **P < 0.01, ***P < 0.001.

We further investigated the effect of UNC0379 on renal tubular cell injury and apoptosis in the murine model of cisplatin-induced AKI. As indicated by IF and immunoblot analysis, expression of neutrophil gelatinase-associated lipocalin (NGAL), a biomarker of kidney injury, was induced in the renal tubules of cisplatin-treated mice; UNC0379 administration totally blocked its expression (Fig. 2A, C, D). The TdT-mediated dUTP nick-end labeling (TUNEL) assay also displayed an increased number of TUNEL (+) cells in cisplatin-alone treated kidneys compared with vehicle-treated kidneys, and UNC0379 treatment largely reduced this population (Fig. 2A, B). In parallel, cisplatin enhanced the level of cleaved caspase 3 (Cleaved cas3), a major marker of apoptosis in the kidney; caspase 3 was reduced to the basal levels in the presence of UNC0379 (Fig. 2C, E). In contrast, cisplatin treatment led to downregulation of E-cadherin and PTEN; application of UNC0379 not only restored but also enhanced expression of PTEN, but this treatment did not increase E-cadherin expression (Fig. 2F–H). IF staining illustrated that PTEN was extensively expressed in renal tubules of the vehicle or UNC0379-alone treated animals; cisplatin treatment remarkedly reduced PTEN expression, while UNC0379 restored its expression (Fig. 2I). Notably, PTEN was primarily located in the cytoplasm of renal tubules of the vehicle or UNC0379-alone treated animals. Cisplatin increased PTEN distribution in the nucleus, and UNC0379 diminished this response. These data suggest that SET8 activation is critically involved in renal tubular cell injury and apoptosis and is required for PTEN suppression and redistribution of PTEN in the nucleus of injured proximal tubular cells.

### Cisplatin induces SET8 and H4K20me1 expression, promotes caspase 3 cleavage, and represses PTEN expression in cultured TKPTs

**Fig. 3:**
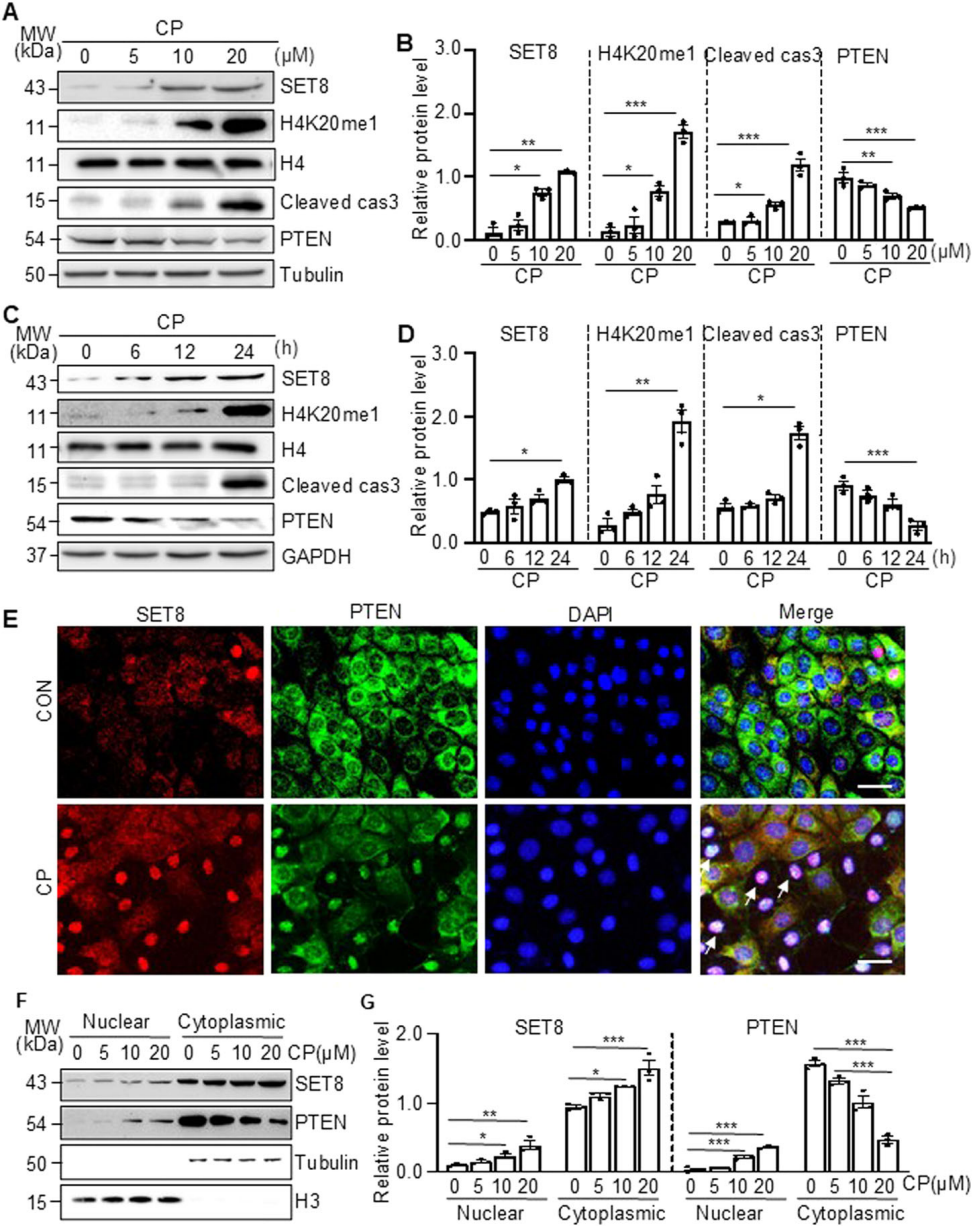
Cisplatin (CP) induced SET8 and H4K20me1 expression, caspase 3 cleavage, and co-localization of SET8 and PTEN in the nucleus of TKPTs. Mouse proximal tubule cells (TKPTs) were exposed to various concentrations (0, 5, 10, and 20 μM) of CP for 24 h (**A**, **B**) or treated with cisplatin (20 μM) for 0, 6, 12, and 24 h (**C**, **D**). Cell lysates were prepared and subjected to immunoblot analysis against SET8, H4K20me1, histone H4 (H4), cleaved cas3, PTEN, Tubulin, or GAPDH. The expression levels of all those proteins were quantified by densitometry. SET8, cleaved cas3, and PTEN in Fig. 3A were normalized by Tubulin (**B**), and those protein levels in Fig. 3C were normalized by GAPDH (**D**); H4K20me1 in Fig. 3A, C, was normalized by H4 (**B**, **D**). IF staining for SET8 and PETN is indicated (**E**). DAPI staining was used to localize nuclei in TKPT. Scale bars = 20 μm. Arrows: nuclei with SET8 and PETN staining. **F** Western blotting and quantification (**G**) of the nuclear and cytoplasmic distribution of SET8 and PTEN at various concentrations of cisplatin. Histone H3 (H3) and Tubulin serve as nuclear and cytoplasmic markers, respectively. Data are represented as the mean ± SEM of at least three experiments. *P < 0.05, **P < 0.01, ***P < 0.001.

To confirm the role of SET8 in the cisplatin-induced apoptosis of renal tubule cells in vitro, we first examined the expression of SET8 and H4K20me1, along with caspase 3 cleavage and PTEN expression, in cultured TKPTs following cisplatin exposure. As shown in Fig. 3A, B, exposure of TKPTs to cisplatin led to a dose-dependent increase of SET8 and H4K20me1, with a significant increase observed at a dose of 10 µM and a further increase at 20 µM. The time course study with 20 µM of cisplatin demonstrated a significant increase of SET8 and H4K20me1 at 24 h (Fig. 3C, D). Coincident with the upregulation of SET8 and H4K20me1, Cleaved cas3 was increased, and the expression of PTEN was reduced in a dose-dependent and time-dependent manner, with significant change at a cisplatin dose of 20 µM (Fig. 3A, B) and 24 h (Fig. 3C, D). IF staining indicated that SET8 and PTEN were mainly located in the cytoplasm of normally cultured TKPTs, and both were clearly observed in the nucleus of some cells following cisplatin treatment (Fig. 3E). Along with this, immunoblot analysis of the proteins extracted from the cytoplasm and nuclei showed a large amount of SET8 and PTEN in the cytoplasm and a small portion of them in the nucleus of TKPTs. Moreover, the induction of the nuclear location of SET8 and PTEN was dependent on the cisplatin dose. Significant increases of nuclear SET8 and PTEN were induced by 10–20 μM. Of note, cisplatin treatment dose-dependently increased both the cytoplasmic and nuclear SET8 and nuclear PTEN, accompanied by reduced cytoplasmic PTEN (Fig. 3F, G). These data, together with in vivo observations, suggest that cisplatin induces activation of SET8, which is accompanied by downregulation and nuclear translocation of PTEN and activation of caspase 3.

### Inhibition of SET8 by UNC0379 or siRNA silencing reduces cisplatin-induced apoptosis in cultured TKPTs

**Fig. 4:**
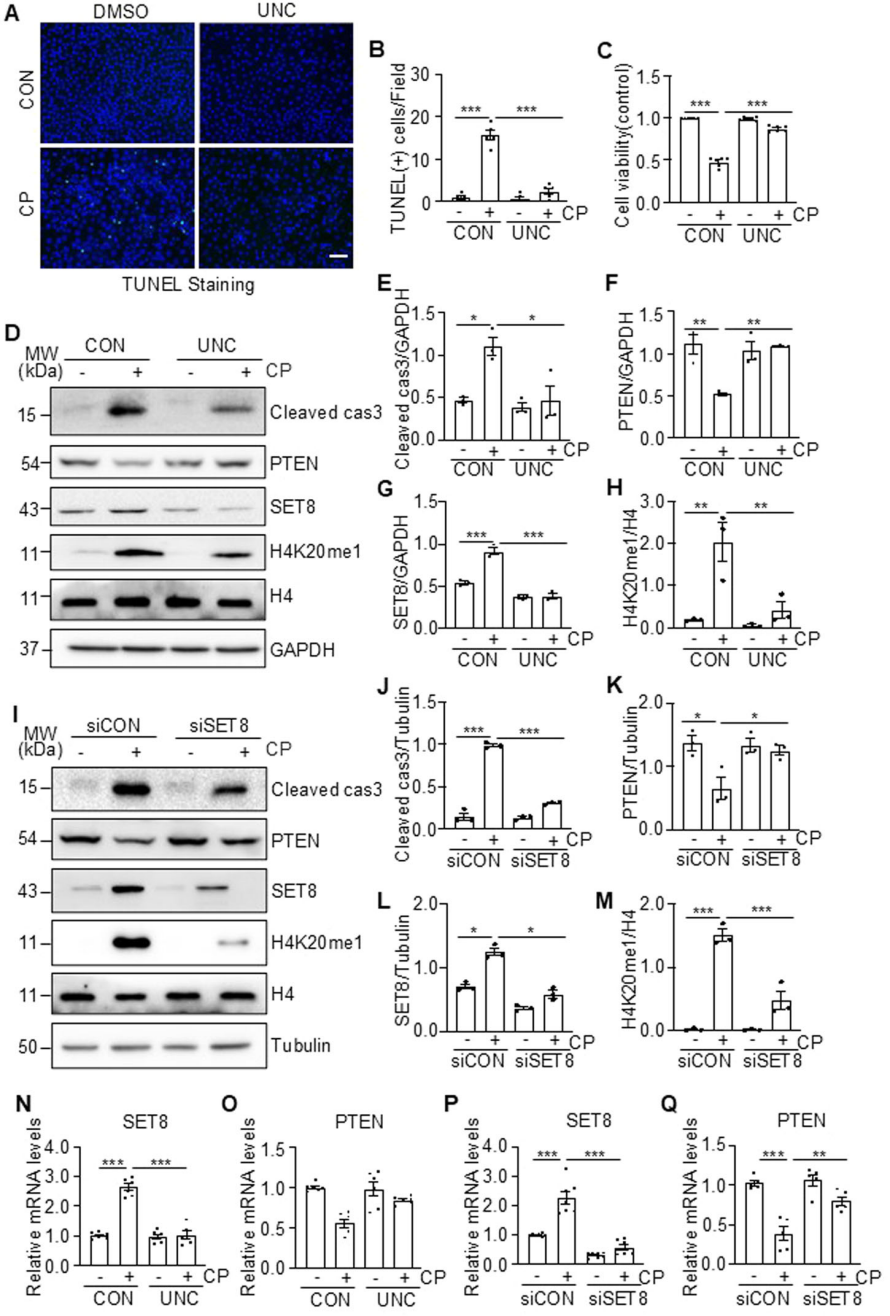
SET8 inhibition reduces cisplatin (CP)-induced apoptosis, along with preservation of PTEN in TKPTs. TKPTs were treated with UNC0379 for 1 h (**A**–**H**) or transfected with siRNA targeting SET8 (siSET8) or control siRNA (siCON) (I-M) for 24 h and then exposed to cisplatin for 24 h. **B** Cells were conducted for TUNEL staining (**A**), and TUNEL (+) cells are shown. DAPI staining was used to localize nuclei in TKPTs. Scale bars = 50 μm. **C** Cell viability was detected after 24 h by cell counting kit 8 (CCK8). Cell lysates were prepared and subjected to immunoblot analysis with antibodies as indicated (**D**, **I**). The expression levels of all the proteins were quantified by densitometry. Cleaved cas3 (**E**), PTEN (**F**), and SET8 (**G**) were normalized by GAPDH; H4K20me1 (**H**) was normalized by H4; Cleaved cas3 (**J**), PTEN (**K**), and SET8 (**L**) were normalized by Tubulin; H4K20me1 (**M**) was normalized by H4. Cells were treated as described above. The extracted RNA cell lysates were subjected to quantitative RT-PCR using the primers listed in Supplementary Table 3. mRNA expression levels of SET and PCNA were normalized relative to mouse β-Actin and analyzed using the 2^−ΔΔCt^ method (**N**–**Q**). Data are represented as the mean ± SEM of at least three experiments. *P < 0.05, **P < 0.01, ***P < 0.001.

To investigate the role of SET8 in regulating renal tubular cell apoptosis and PTEN expression in culture, we first examined the effect of UNC0379 on cisplatin-induced cellular apoptosis, cleaved cas3, and expression of PTEN and E-cadherin. As shown in Fig. 4A–C, treatment with UNC0379 mitigated cisplatin-induced apoptosis, as evidenced by a reduced number of TUNEL-positive cells and increased cell survival. UNC0379 also decreased expression levels of cleaved cas3 (Fig. 4D, E) and restored expression of PTEN but did not affect CP-induced downregulation of E-cadherin (Fig. 4D, F, and Supplementary Fig. 2A, B). UNC0379 reduced the cisplatin-induced expression of SET8 and H4K20me1 without altering the expression level of total histone 4 (Fig. 4D, G, H), indicating the effectiveness of this inhibitor. Next, we examined the effect of siRNA specifically designed for SET8 on the apoptosis of cisplatin-induced renal tubular cell apoptosis. Silencing SET8 also effectively suppressed expression levels of cleaved cas3 (Fig. 4I, J), reduced the number of TUNEL-positive cells (Supplementary Fig. 1A, B), and enhanced cell viability (Supplementary Fig. 1C) in TKPTs exposed to cisplatin. Moreover, transfection of SET8 siRNA largely reduced the expression level of SET8 and H4K20me1 and preserved the expression of PTEN (Fig. 4I, K–M) but did not affect CP-induced downregulation of E-cadherin (Supplementary Fig. 2C, D). In addition, both treatment with UNC and transfection with SET8 siRNA retained the mRNA expression of PTEN in TKPTs following exposure to cisplatin (Fig. 4N–Q). These results indicated that SET8 activation is critically involved in cisplatin-induced apoptosis and the downregulation of PTEN in renal tubular cells.

### SET8 mediates cisplatin-induced DDR in the kidney and cultured TKPTs

**Fig. 5:**
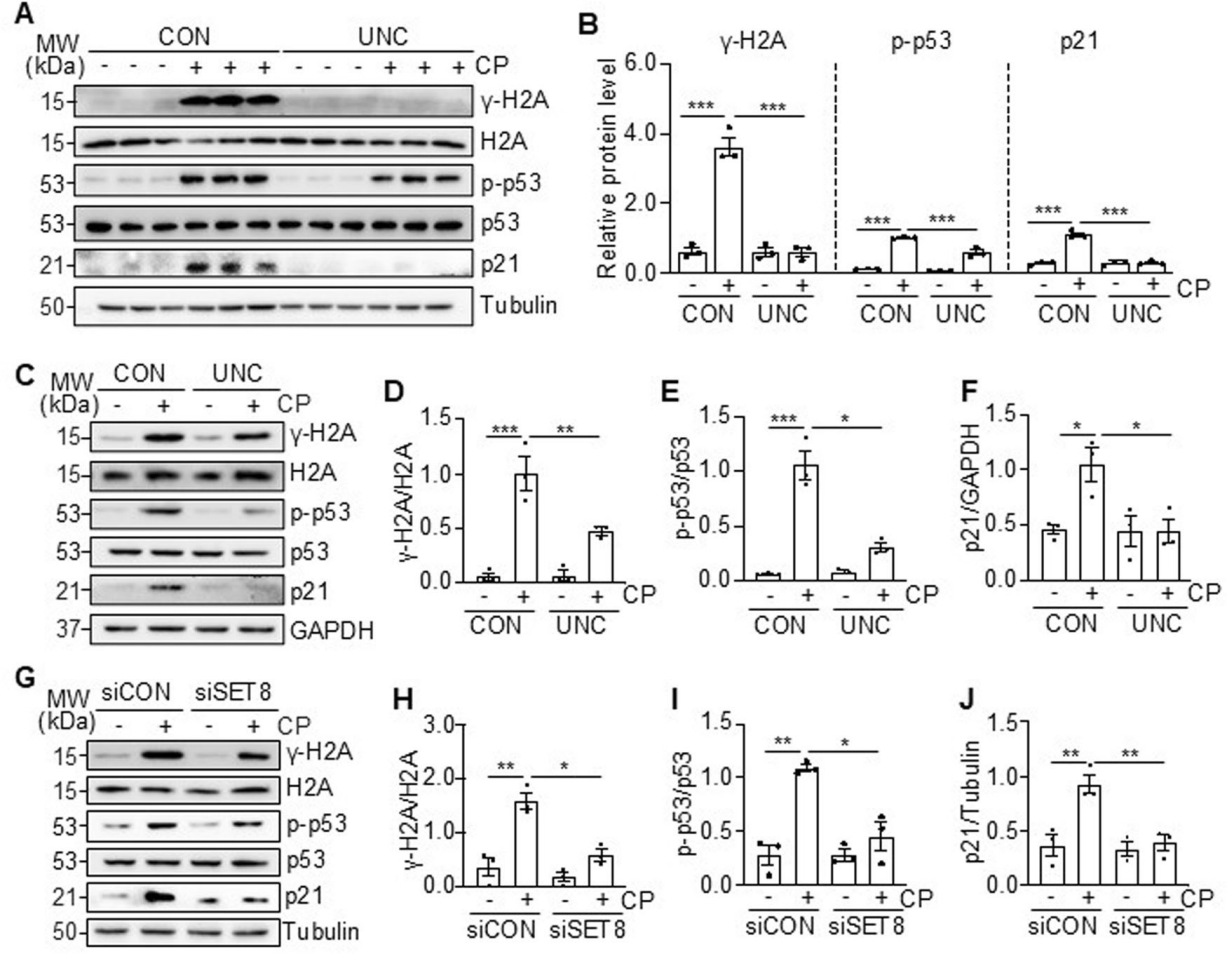
Inhibition of SET8 reduces DDR in the kidney and TKPTs following cisplatin (CP) treatment. Mice were treated with cisplatin and UNC0379 (UNC) as indicated in Fig. 1A. Kidney tissue lysates were prepared and subjected to immunoblot analysis using antibodies as indicated (**A**). The levels of all the proteins were quantified by densitometry, and γ-H2A, p-p53, and p21 were normalized with H2A, p53, and Tubulin, respectively, (**B**). TKPTs were treated with UNC0379 for 1 h (**C**–**F**) or transfected with siRNA targeting SET8 (siSET8) or control siRNA (siCON) (**G**–**J**) for 24 h and then exposed to cisplatin for 24 h. Cell lysates were prepared and subjected to immunoblot analysis using antibodies as indicated (**C**, **G**). The levels of all the proteins were quantified by densitometry. γ-H2A (**D**), p-p53 (**E**), and p21 (**F**) were normalized with H2A, p53, and GAPDH, respectively, and γ-H2A (**H**), p-p53 (**I**), and p21(**J**) were normalized with H2A, p53, and Tubulin, respectively. Data are represented as the mean ± SEM of at least three experiments. *P < 0.05, **P < 0.01, ***P < 0.001.

DNA damage is the primary event of renal tubular cell apoptosis, and SET8 has been reported to be associated with DDR in tumor cells [21]. To understand whether SET8-induced renal tubular cell apoptosis is associated with DDR following cisplatin treatment, we investigated the effect of SET8 inhibition on the phosphorylation of H2AX (γ-H2AX) and p53 (p-p53) and the expression of p21 in vivo and in vitro [5]. In the kidney of mice, cisplatin treatment led to increased expression of γ-H2AX, p-53, and p21, UNC0379 administration significantly reduced γ-H2AX and p21 expression and phosphorylated p53 level (Fig. 5A, B). Similarly, in the cultured TKPTs, exposure to cisplatin resulted in increases of all these protein levels, and UNC0379 treatment was also effective in reducing their expression and phosphorylation levels (Fig. 5C–F). Similar results were observed in TKPTs transfected with SET8 siRNA and exposed to cisplatin as well (Fig. 5G–J). Therefore, we suggest that SET8 activation is necessary for the induction of DDR by cisplatin in the kidney and renal epithelial cells.

### SET8 mediates the impairment of autophagy in the kidney and cultured TKPTs following cisplatin treatment

**Fig. 6:**
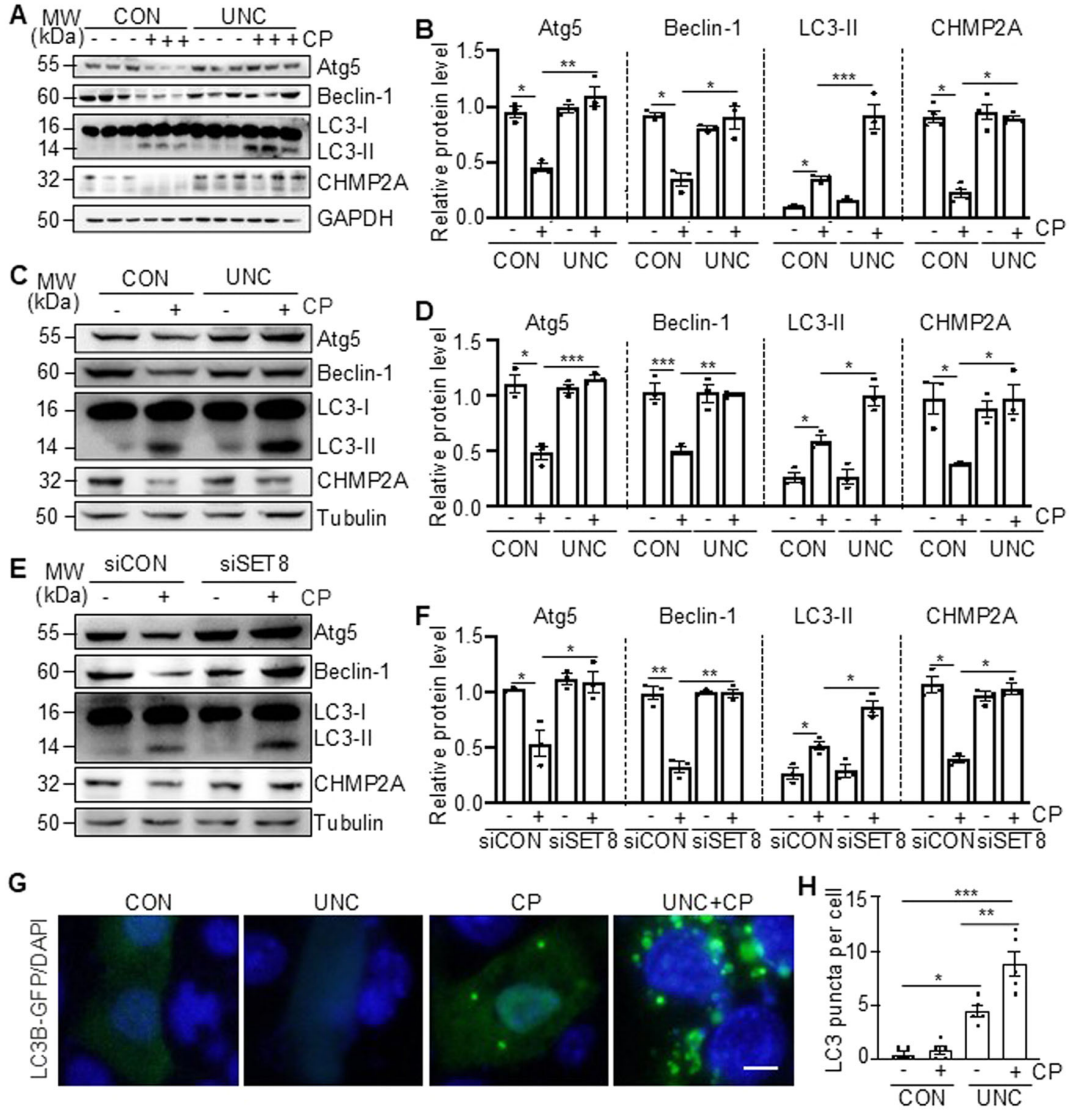
Inhibition of SET8 restores the autophagic response in the kidney and TKPTs following cisplatin (CP) treatment. Mice were treated with CP and UNC0379 (UNC) as indicated in (**A**). Kidney tissue lysates were subject to immunoblot analysis using antibodies against Atg5, Beclin-1, LC3-I/LC3-II, Charged Multivesicular Body Protein 2A (CHMP2A), GAPDH. The levels of all those proteins were quantified by densitometry and individually normalized with GAPDH (**B**). TKPTs were pretreated with UNC0379 for 1 h (**C**, **D**) or transfected with control siRNA or SET8 siRNA for 24 h (**E**, **F**) and then exposed to CP for 24 h; cell lysates were prepared and subjected to immunoblot analysis using antibodies as indicated (**C**, **E**). The levels of all the proteins were quantified by densitometry and then individually normalized with Tubulin (**D**, **F**). TKPTs were transfected with LC3B-GFP expression plasmid and then treated with CP for 24 h in the presence or absence of UNC (**G**, **H**). DAPI staining was used to localize nuclei in TKPTs. The number of LC3B-GFP puncta per field was quantified (**H**). Scale bars = 5 μm. Data are represented as the mean ± SEM of at least three experiments. *P < 0.05, **P < 0.01, ***P < 0.001.

In contrast to DNA damage, autophagy plays a protective role in AKI and renal tubular apoptosis induced by cisplatin [4]. Here, we further investigated the possible involvement of SET8 in autophagy by examining the expression of several molecules required for the formation of autophagosomes, including Atg5, Beclin-1, CHMP2A, and LC3-I/II. As shown in Fig. 6A, B, Atg5, Beclin-1, and CHMP2A were abundantly expressed in control kidneys and dramatically reduced following cisplatin treatment; administration of UNC0379 restored their expression. An abundance of LC3-I, but not LC3-II (the autophagic form of LC3), was also detected in control kidneys. Cisplatin induced an obvious accumulation of LC3-II, which was further increased upon UNC0379 treatment, however, UNC0379 did not significantly increase LC3-II expression in control kidneys. Similarly, treatment with either UNC0379 or SET8 siRNA also preserved the expression of Atg5, Beclin-1, and CHMP2A and enhanced levels of LC3-II in TKPTs following cisplatin exposure (Fig. 6C–F). In addition, silencing of SET8 with siRNA was effective in suppressing the cisplatin-induced increased expression of p62, another marker of autophagy that is induced to be degraded by autophagy (Supplementary Fig. 3A–C).

Since redistribution of LC3 from the cytosol to a punctate autophagosome staining is an indication of autophagy, we also assessed the effect of UNC0379 on LC3 distribution in TKPTs. In the end, TKPTs were transfected with a green fluorescent protein (GFP)-LC3 fusion plasmid and then treated with cisplatin for 24 h, followed by examination by fluorescence microscopy. In the control TKPTs, very few transfected (GFP-labeled) cells had punctate LC3 staining; cisplatin treatment increased the percentage of LC3 punctate cells to 40% (Fig. 6G, H).

Given that autophagy induction is a defense response against apoptosis induced by cisplatin, these data suggest that SET8 activation may contribute to renal epithelial cell apoptosis, at least in part by abrogating the autophagic prosurvival response.

### Cisplatin-induced PTEN downregulation is required for SET8 to induce DDR and apoptosis in renal epithelial cells

**Fig. 7:**
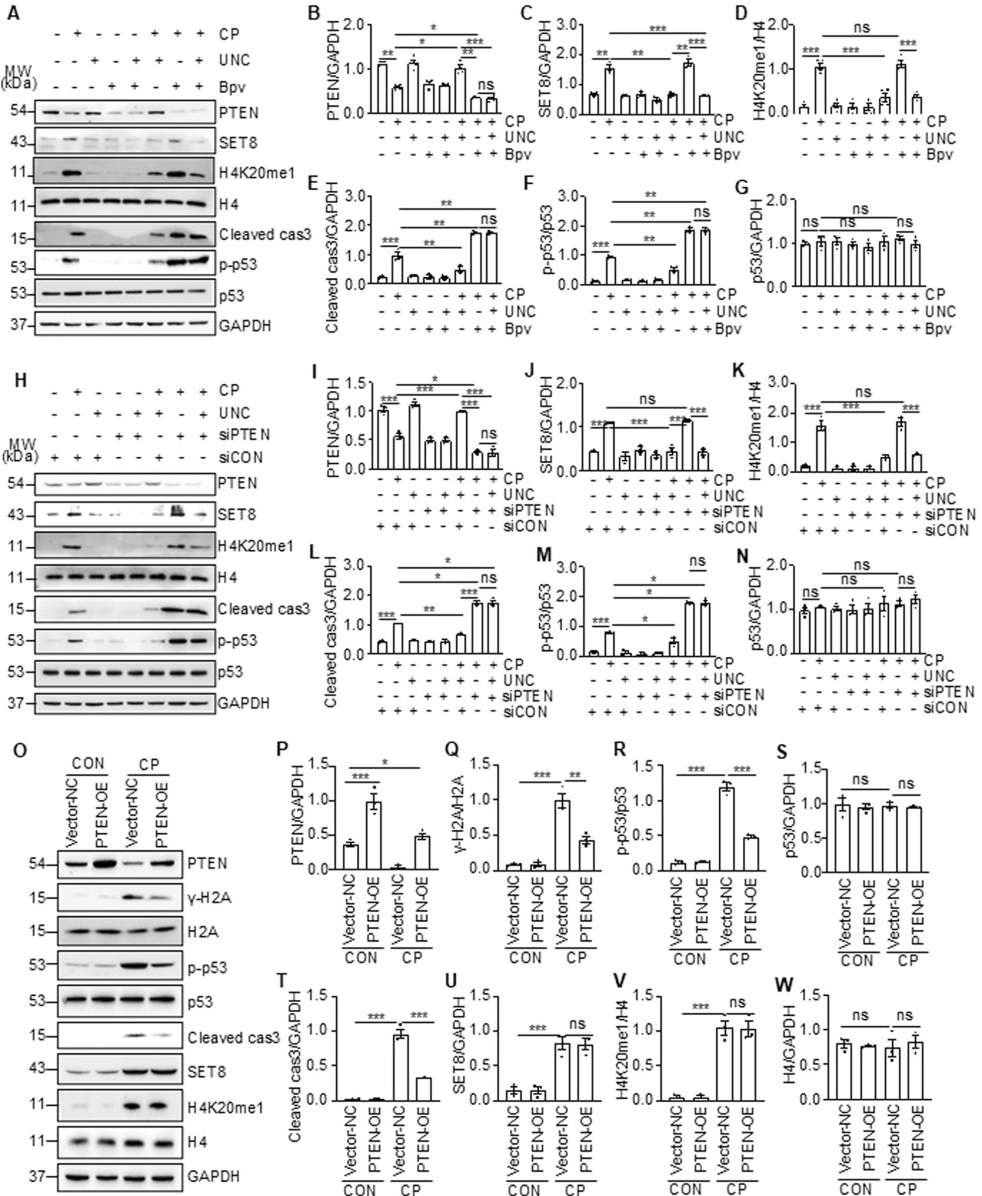
Inhibition of PTEN promoted cisplatin (CP)-induced apoptosis and DDR, and conversely, alleviated by overexpression of PTEN in TKPTs. TKPTs were treated with CP for 24 h in the presence or absence of UNC and Bpv as indicated (**A**) or after transfection of siRNA targeting PTEN (siPTEN) or control siRNA (siCON) for 24 h (**H**). Cell lysates were prepared and subjected to immunoblot analysis with antibodies as indicated. Expression levels of all the proteins were quantified by densitometry, and PTEN (**B**, **I**), SET8 (**C**, **J**), Cleaved cas3 (**E**, **L**) and p53 (**G**, **N**) were normalized with GAPDH, H4K20me1 (**D**, **K**) was normalized with H4, and p-p53 (**F**, **M**) was normalized with p53. TKPTs were transfected with PTEN plasmid (PTEN-OE) or empty vector (Vector-NC) and then exposed to CP for an additional 24 h. Cell lysates were prepared and subjected to immunoblot analysis with antibodies as indicated (**O**). Expression levels of all the proteins were quantified by densitometry, and PTEN (**P**), Cleaved cas3 (**T**), SET8 (**U**), p53 (**S**) and H4 (**W**) were normalized with GAPDH; γ-H2A (**Q**), p-p53 (**R**), and H4K20me1 (**V**) were normalized with H2A, p53, and H4, respectively. Data are represented as the mean ± SEM of at least three experiments. *P < 0.05, **P < 0.01, ***P < 0.001.

To understand the relationship between PTEN expression and DNA damage and apoptosis, we further examined the effect of bpV HOpic (Bpv), an efficient PTEN-specific inhibitor [22] and a siRNA specifically silencing PTEN on cisplatin-induced apoptosis and phosphorylation of p53 and H2AX in cultured TKPTs. In agreement with the above observations, cisplatin reduced the expression of PTEN and increased the expression of SET8, H4K20me1, p-p53, and cleaved cas3; UNC0379 restored the expression of PTEN and reduced the expression of p-p53 and cleaved cas3 (Fig. 7A–G). Interestingly, treatment with either Bpv or PTEN siRNA in the absence of UNC0379 potentiated cisplatin-induced expression of p-p53 and cleaved cas3 but did not affect expression of SET8 and H4K20me1; inhibition of PTEN with Bpv or siRNA suppressed UNC0379-elicited restoration of PTEN and abolished the inhibitory effect of UNC0379 on the expression of p-p53 and cleaved cas3 in TKPTs exposed to cisplatin (Fig. 7H–N). As γ-H2AX is an early event in response to DNA damage and PTEN was reported to be able to dephosphorylate γ-H2AX [13], we also examined the effect of SET8 inhibition on the expression of γ-H2AX. As shown in Supplementary Fig. 4A–D, inhibition of PTEN with Bpv or its siRNA increased cisplatin-induced expression of γ-H2AX compared with those treated with vehicle or control siRNA. These data suggest that PTEN preservation is vital for SET8 inhibition to antagonize DNA damage and apoptosis.

To verify the role of PTEN in cisplatin-induced DDR and apoptosis and in relation to SET8, we investigated the effect of PTEN overexpression on the phosphorylation of H2AX and p53 as well as the cleavage of caspase 3. Figure 7O–W demonstrated an increase of PTEN expression in TKPTs transfected with PTEN plasma vector compared with those transfected with control vectors; cisplatin treatment remarkably reduced PTEN expression in control vector transfecting cells and reduced its expression in PTEN transfecting cells. PTEN overexpression did not affect expression of SET8 and H4K20me1 levels, but significantly reduced expression levels of γ-H2AX and p-53 and cleaved cas3.

In summary, these data support the notion that SET8 induces apoptosis through a mechanism associated with repression of PTEN and consequent induction of DDR.

### Cisplatin-induced downregulation of PTEN contributes to the impairment of autophagy and potentiates apoptosis in renal epithelial cells

**Fig. 8:**
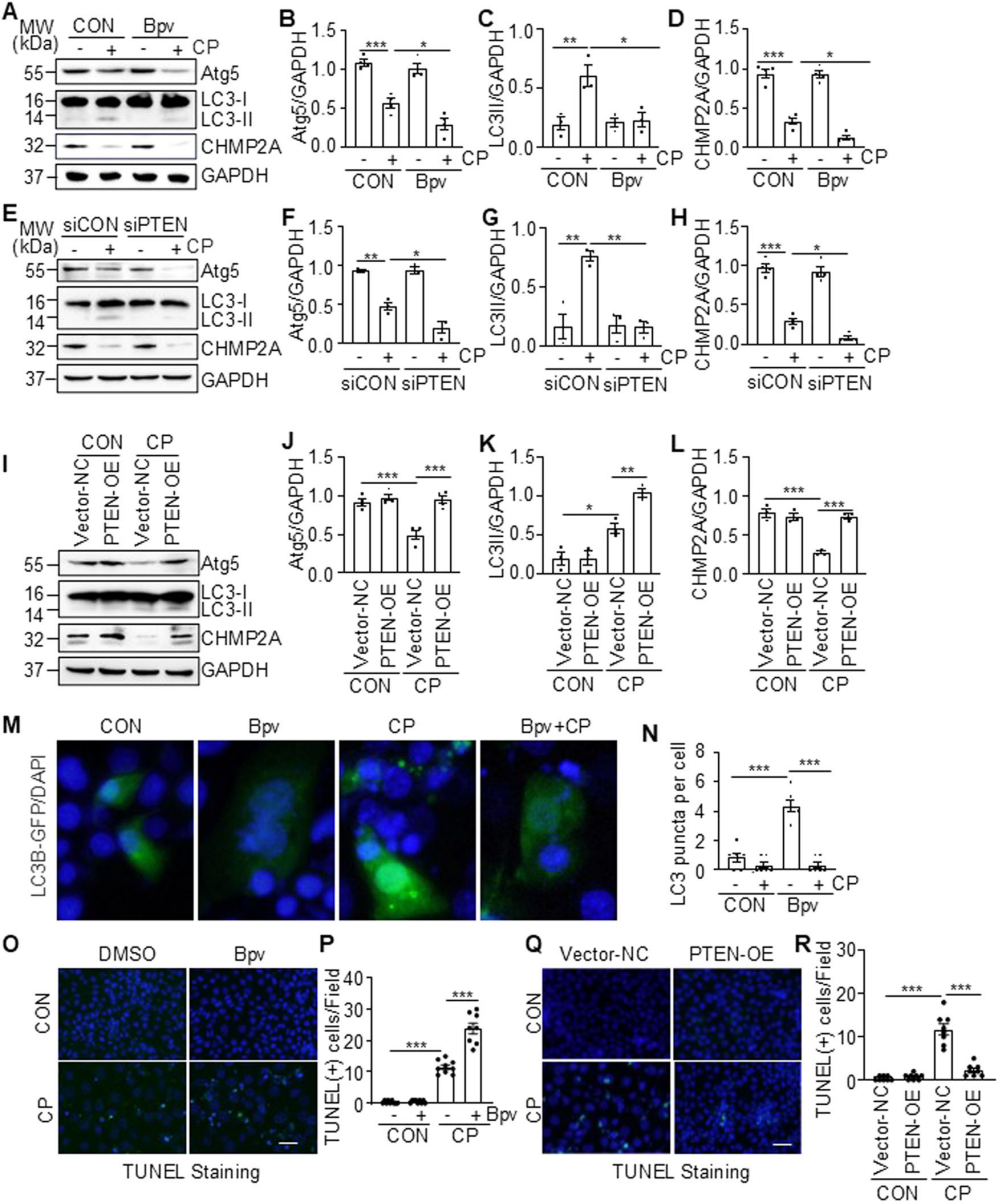
Inhibition of PTEN potentiates cisplatin (CP)-induced impairment of autophagy, and conversely, is preserved by overexpression of PTEN in TKPTs. TKPTs were treated with CP for 24 h in the presence or absence of Bpv (**A**) or after transfection of siRNA targeting PTEN (siPTEN) or control siRNA (siCON) for 24 h (**E**). Cell lysates were prepared and subjected to immunoblot analysis using antibodies as indicated (**A**, **E**). The levels of all the proteins were quantified by densitometry and then individually normalized with GAPDH and LC3-I, respectively (**B**–**D**, **F**–**H**). TKPTs were transfected with PTEN plasmid (PTEN-OE) or empty vector (Vector-NC) for 24 h and then exposed to CP for an additional 24 h (**I**). Cell lysates were prepared and subjected to immunoblot analysis with antibodies as indicated (**I**) and then individually normalized with GAPDH (**J**, **L**) or LC3I-I (**K**). TKPTs were transfected with the LC3B-GFP plasmid and then treated with CP for 24 h in the presence or absence of UNC (**M**, **N**). Cells were photographed with a fluorescent microscope (**M**). The number of LC3B-GFP puncta per field was quantified (**N**). Scale bars = 5 μm TKPTs were treated with Bpv (**O**, **P**) or transfected with PTEN plasmid (PTEN-OE) or empty vector (Vector-NC) (**O**–**R**) for 24 h and then exposed to CP for an additional 24 h. Cells were conducted for TUNEL staining (**O**, **Q**), and the number of TUNEL (+) cells per field was counted in at least 10 fields per section (**P**, **R**). Scale bars = 25 μm. Data are represented as the mean ± SEM of at least three experiments. *P < 0.05, **P < 0.01, ***P < 0.001.

We proceeded to investigate the role of PTEN in the regulation of autophagy in renal epithelial cells following cisplatin treatment by PTEN suppression and overexpression. Suppression of PTEN with either Bpv or siPTEN further reduced expression of Atg5, LC3-II, and CHMP2A (Fig. 8A–H and Supplementary Fig. 5A–D), while overexpression of PTEN restored expression of Atg5 and CHMP2A and enhanced the expression of LC3-II in TKPTs exposed to cisplatin (Fig. 8I–L). Figure 8M, N further demonstrated that blocking PTEN by Bpv reduced cisplatin-induced LC3 punctate formation in TKPTs. Additionally, treatment with Bpv enhanced while overexpression of PTEN reduced apoptosis of renal tubular cells following exposure to cisplatin (Fig. 8O–R). These data, together with the role of SET8 in downregulation of PTEN shown in Figs. 2, 4 and 7 as well as the reported protective role of autophagy in renal epithelial cells following cisplatin exposure [7], suggest that SET8-mediated loss of PTEN leads to the impairment of autophagy and enhancement of renal tubular cell apoptosis in response to cisplatin treatment.

### Treatment with chloroquine reverses the enhancement of autophagy and the inhibition of apoptosis induced by SET8 inhibition in renal tubular cells

**Fig. 9:**
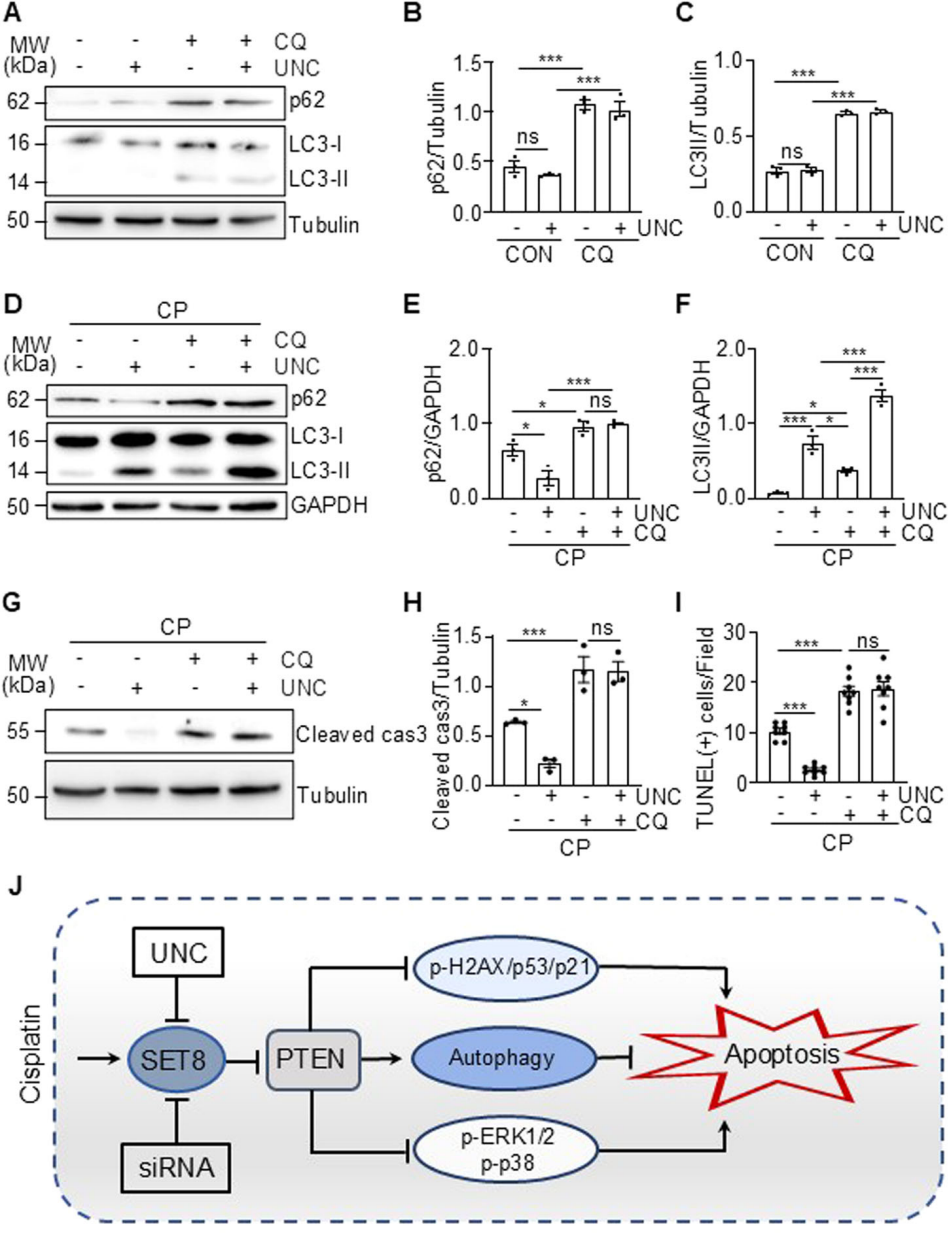
Treatment with chloroquine reverses the enhancement of autophagy and the inhibition of apoptosis induced by SET8 inhibition in renal tubular cells. TKPTs were treated with vehicle (DMSO) or CP (20 μM) for 24 h in the presence or absence of 10 μM chloroquine (CQ) and 20 μM UNC0379 (UNC) as indicated in the figures. Cell lysates were prepared and subjected to immunoblot analysis using antibodies as indicated (**A**, **D**, **G**). The expression levels of all the proteins were quantified by densitometry and then individually normalized with tubulin (**B**, **C**, **H**) or GAPDH (**E**, **F**). The number of TUNEL-positive cells per field was counted in at least 10 fields per section (**I**). Data are represented as the mean ± SEM of at least three experiments. *P < 0.05, **P < 0.01, ***P < 0.001. Schematic diagram for the role of SET8 and PTEN in CP-induced renal tubular cell apoptosis and acute kidney injury (**J**).

We further employed chloroquine, an autophagy inhibitor, to investigate whether the inhibition of SET8 would enhance autophagic flux and subsequently protect renal tubular cells from apoptosis. Given that chloroquine inhibits autophagy at the autolysosome level [23], it is anticipated to increase p62 expression and LC3-II accumulation. Indeed, we observed that treatment with chloroquine resulted in elevated levels of p62 and increased accumulation of LC3-II compared to those without chloroquine treatment in cultured TKPTs, while treatment with UNC neither significantly altered the expression levels of p62 and LC3-II, nor enhanced chloroquine induced expression of p62 and LC3-II in this cell type (Fig. 9A–C). Figure 9D–F demonstrate that the presence of UNC enhanced autophagic flux in CP-treated cells, as evidenced by reduced expression of p62 and increased levels of LC3-II (comparing Fig. 9D, lane 1 vs lane 2); however, treatment with chloroquine reversed this response (comparing Fig. 9D, lane 2 vs lane 4). As expected, administration of chloroquine alone effectively suppressed CP-induced autophagy, as indicated by increased expression of p62 and LC3-II. Additionally, we examined the effect of chloroquine on CP-induced apoptosis in TKPTs both in the absence and presence of UNC. As illustrated in Fig. 9G–I, chloroquine not only augmented CP-induced apoptosis but also counteracted the inhibitory effect exerted by UNC on apoptosis in TKPTs with CP exposure. Collectively, these findings indicate that the SET8-mediated dysregulation of autophagy is essential for the induction of apoptosis in TKPTs exposed to cisplatin (CP).

### Blocking SET8 inhibits phosphorylation of ERK1/2 and P38 in the kidney and renal epithelial cells following cisplatin treatment

Previous studies have reported that cisplatin-induced activation of p38 and ERK1/2 in the kidney and renal epithelial cells also contributes to apoptosis in renal epithelial cells [24, 25]. Since PTEN can dephosphorylate these two MAPKs through dephosphorylation of Shc, an upstream activator of these two pathways [26, 27], we examined whether SET8 activation is required for the activation of these two pathways. As shown in Supplementary Fig. 6, compared with the control group, cisplatin exposure led to p38 and ERK1/2 phosphorylation both in vitro and in vivo. UNC0379 treatment significantly reduced cisplatin-induced phosphorylation of p38 and ERK1/2 in the kidney of mice and cultured TKPTs. Similarly, silencing SET8 using SET8 siRNA reduced cisplatin-induced p38 and ERK1/2 phosphorylation to the basal levels in TKPTs. In contrast to the above observation, cisplatin did not reduce phosphorylation of AKT in the kidney and renal epithelial cells, and neither UNC0379 nor SET8 siRNA affected AKT phosphorylation (data not shown). These data suggest that SET8 may also contribute to renal cell apoptosis via a mechanism involved in the activation of ERK1/2 and p38 signaling.

## Discussion

SET8 is the lysine methyltransferase involved in the regulation of cell cycle, DNA repair, gene transcription, and other physiological processes [21]. In this study, we uncovered a novel role of SET8 in driving AKI and renal tubular cell apoptosis. Following cisplatin treatment, SET8 and H4K20me1 were upregulated in the kidney of mice with AKI; inhibition of SET8 by UNC0379 improved renal function and attenuated renal tubular damage and apoptosis. Blocking SET8 activation also diminished the cellular apoptosis of cultured renal epithelial cells exposed to cisplatin. Furthermore, cisplatin-induced SET8 activation led to PTEN downregulation and subsequently increased DDR and reduced autophagy in vivo and in vitro. These results suggest that SET8 is a critical driver in the evolution of AKI and renal epithelial cell death following cisplatin treatment and could serve as a potential therapeutic target for nephrotoxic AKI.

To date, studies on SET8 expression and its role in tissue injury and cell death are limited and controversial. While one study shows that SET8 expression is decreased in mice with hepatic ischemia-reperfusion injury and silencing of SET8 with siRNA aggravates the liver injury and inflammation [28], another study indicates that SET8 is highly expressed in acute myeloid leukemia and blocking SET8 promotes apoptosis [29]. Furthermore, the overexpression of SET8 is required for motor function recovery after spinal cord injury in rats [30]. The reason behind the discrepancy is unclear but may be related to different insults and models. In the current study, we demonstrated that following cisplatin treatment, SET8 was upregulated in the kidney and cultured renal epithelial cells and inhibition of SET8 by UNC0379, a specific inhibitor of SET8 or siRNA, attenuated renal injury and tubular cell apoptosis, suggesting that SET8 activation is detrimental to renal epithelial cells in nephrotoxic AKI.

Cisplatin-induced apoptosis is involved in the activation of multiple stress events, including DDR and autophagy [4]. DDR is a signaling pathway activated by DNA double-strand breaks that recruit signaling proteins to the chromatin flanking the lesion to regulate DNA repair, replication stress responses, and apoptosis [5]. Among the numerous proteins involved in DDR, phosphorylation of H2AX is initially required for the assembly of DNA repair proteins and the activation of checkpoint proteins for DNA repair [5]. However, when an injury is severe, the phosphorylated H2AX will activate p53 signaling, leading to DNA replication inhibition, cell cycle arrest, and apoptosis [5]. In this study, we found that cisplatin-induced AKI and renal tubular apoptosis were accompanied by increased phosphorylation of H2AX and p53, expression of p21, and cleavage of caspase 3, while blocking SET8 by either UNC0379 or its specific siRNA inhibited all these responses, suggesting that SET8 mediated DDR contributes to renal epithelial cell death. In contrast to inducing DDR, SET8 activation led to impaired autophagy, which was evidenced by reduced expression of several proteins related to autophagic responses, including Atg5, Beclin-1, and CHMP2A, and increased expression of p62, in vivo and/or in vitro, whereas SET8 inhibition reversed these responses. Moreover, SET8 inactivation increased the expression of LC3-II, a cellular event associated with the formation of autophagosomes, while blocking autophagy with chloroquine effectively reverses the suppression of apoptosis induced by SET8 inhibition in renal tubular cells exposed to cisplatin. Since DNA damage is positively and autophagy is negatively related to cisplatin-induced apoptosis, SET8 activation may drive renal tubular cell apoptosis via a mechanism involved in the upregulation of DDR and downregulation of autophagy in cisplatin nephrotoxicity.

How SET8 is coupled to the DDR and autophagy to promote kidney cell apoptosis remains unclear, but PTEN is critically involved in these processes. Previous studies have shown that normal PTEN expression is required for renal tubular cells to resist the death of renal epithelial cells and promote survival, but its expression is frequently downregulated in the kidney of AKI induced by a variety of insults, including cisplatin [8, 10, 11]. In line with this, the current study showed that PTEN expression levels declined in the kidney and cultured TKPTs following cisplatin treatment, while inhibition of the SET8 activity with UNC0379 restored PTEN expression, along with alleviation of apoptosis both in vivo and in vitro. Moreover, blocking PTEN expression abolished the anti-apoptotic effects of SET8 inhibition. Since suppression of PTEN aggravated cisplatin-induced DDR and lowered autophagic response, and overexpression of PTEN resulted in an opposite effect, these data support the idea that PTEN loss is necessary for transferring the SET8 signal to DNA damage and autophagy dysfunction. In this context, it has been reported that reduced expression of PTEN in human embryonic kidney cells also promoted DNA damage [31], and PTEN deficiency in murine kidneys inhibited phagosome closure and autolysosome formation after ischemia-reperfusion by reducing expression of CHMP2A, a protein necessary for autolysosome formation [10]. In this study, we found that CHMP2A was suppressed in cisplatin nephrotoxicity and restored with overexpression of PTEN, in favor of the regulatory role of PTEN in autophagy. In addition, studies have shown that nuclear PTEN can directly dephosphorylate the γ-H2AX, leading to abnormal DNA repair [13], which corresponds to our observations that upon cisplatin treatment, PTEN was translocated from the cytosol to the nuclei, where it co-localizes with SET8 in renal epithelial cells.

The mechanism by which SET8 activation leads to loss of PTEN in the kidney needs to be explored. It is well known that PTEN expression and activation are subjected to transcriptional and posttranscriptional regulation [12]. A recent study shows that FOXO1, a member of the forkhead family of transcription factors, occupies the PTEN promoter region in human umbilical vein endothelial cells, and silencing SET8 enhances the role of FOXO1 in promoting activation of the PTEN promoter [32]. Although FOXO1 expression levels were reduced in the injured kidney and renal epithelial cells, administration of either UNC0379 or a siRNA specifically silencing SET8 did not restore FOXO1 expression (data not shown), suggesting that FOXO1 may not play a major role in the cisplatin-induced downregulation of PTEN by SET8. Another possibility is that SET8- induces silencing of PTEN genes by its regulation on H4K20me1; however, it has been reported that SET8-driven H4K20me1 mostly facilitates chromatin openness and accessibility rather than chromatin closeness and inaccessibility [21]. As such, SET8 may methylate other unidentified proteins to mediate its effects on transcription. In addition, SET8 may regulate PTEN expression at the posttranslational level. In this context, SET8 was reported to regulate DNA methyltransferase 1 and its accessory factor, UHRF1 (Ubiquitin Like With PHD And Ring Finger Domains 1), through methylation-dependent protein degradation [33]. Since ubiquitination can regulate the catalytic activity and degradation of PTEN in other systems [34], we cannot rule out the possibility that SET8 directly methylates PTEN, leading to its ubiquitination and degradation. Further investigations are required to address this issue.

Mounting evidence has highlighted the role of the mitogen-activated protein kinase (MAPK) signaling pathway, in particular ERK1/2 and p38, in mediating cisplatin-induced AKI and renal epithelial cell apoptosis [35–37]. In this study, we found that blocking SET8 also inhibited phosphorylation (activation) of these two enzymes in vivo and in vitro models of AKI induced by cisplatin. Previous studies have demonstrated that cisplatin-induced ERK1/2 activation can directly phosphorylate p53, leading to the upregulation of cell cycle arrest and DNA damage [38], and p38 contributes to cisplatin-induced apoptosis through a mechanism associated with the activation of caspase 3 [39]. Thus, SET8-mediated activation of the ERK1/2 and p38 pathways may also be necessary for inducing renal tubular cell apoptosis by cisplatin. SET8 regulation of these two pathways may be through a mechanism associated with PTEN. As a dual-specific phosphatase, PTEN can dephosphorylate many phosphorylated serine, threonine, and tyrosine proteins, including Shc [27, 40]. Since Shc is a common upstream activator of ERK1/2 [41] and p38 [26] and also mediates cisplatin-induced apoptosis of renal epithelial cells [42], it is speculated that SET8-mediated PTEN downregulation reduces its dephosphorylation action on Shc, thereby increasing the phosphorylation (activation) of ERK1/2 and p38 in cisplatin treated kidney and cultured TKPTs.

In summary, our study was the first to demonstrate the critical role of SET8 in driving cisplatin-induced AKI and renal epithelial cell apoptosis. Mechanically, SET8-induced apoptosis may be associated with PTEN loss mediated promotion of DDR and inhibition of autophagy, as well as activation of ERK1/2 and p38 signaling pathways (Fig. 9J). Since SET8 has been implicated in the development of a variety of cancers and the widespread use of cisplatin as a chemotherapeutic agent for most solid tumors, but with known nephrotoxicity, the results from this study suggest that combined administration of SET8 inhibitors with cisplatin could enhance tumor eradication while simultaneously protecting against AKI.

## Materials and methods

### Reagents and antibodies

UNC0379 (S7570) and Bpv (S8651) were purchased from Selleck Chemicals (Huston, TX, USA). PFA (HY-15484) was purchased from MedChemExpress (Monmouth, NJ, USA). TUNEL kit (11684795910) and CCK-8 assay (96998) were purchased from Sigma–Aldrich (St. Louis, MO, USA). Cisplatin was purchased from the pharmacy of Rhode Island Hospital (NDC 0703-5747-11, RI, USA). PTEN siRNA (4390771), Lipofectamine 3000 (L3000001), and SuperSignal chemiluminescent substrate (34580) were purchased from Thermo Fisher Scientific (Waltham, MA, USA). RIPA lysis buffer (9806) was purchased from Cell Signaling Technology (Danvers, MA, USA). con siRNA (sc-37007), SET8 siRNA (sc-155946), and p53 siRNA (sc-29436) were purchased from Santa Cruz Biotechnology (Dallas, TX, USA). The primary antibodies utilized for Western blotting were documented in Supplementary Table 1.

### Mouse models of AKI and treatment

Male C57BL/6J mice (20–25 g) aged 6–8 weeks were purchased from the Jackson Laboratory (Bar Harbor, ME, USA). The mice were randomized into four groups with 6 mice for each group. The sample size was determined based on power calculations, estimating *n* = 6 per group to detect a 50% difference with a power of 0.80 and an alpha of 0.05. The mice were intraperitoneally injected with cisplatin at 20 mg/kg to induce AKI. UNC0379, dissolved in a solvent comprising 10% DMSO and 90% sterile corn oil, was injected 2 h before cisplatin administration at a dose of 5 mg/kg and daily for three consecutive days. The control and cisplatin groups were injected with an equal volume of saline. Mice were euthanized on day 3 after cisplatin injection. Blood samples and kidney tissues were collected for further analysis. All the experimental procedures were approved by Lifespan Animal Welfare Committee at Rhode Island Hospital (USA).

### Renal function analysis

SCr and BUN levels were detected using Creatinine Assay kit (MAK080) and BUN assay kit (MAK006) from Sigma–Aldrich (St. Louis, MO, USA), respectively, according to manufacturer’s instructions.

### Histology and immunofluorescence staining

Histology and immunofluorescence staining procedures are described in our previous studies [6]. Renal tubular damage was evaluated according to Paller’s method [43]. Briefly, five fields were randomly observed, and morphological damage (epithelial necrosis, luminal necrotic debris, and tubular dilation) was quantified using the following scale: none = 0; <10% = 1; 11–25% = 2; 26–75% = 3; and >75% = 4. Nuclei were stained with DAPI (H-1500, Newark, Vector, CA, USA). Fluorescence was visualized and photographed under fluorescence microscopy (Olympus, Shinjuku-ku, Tokyo, Japan). The antibodies employed for immunofluorescence staining were listed in Supplementary Table 2.

### Detection of apoptosis

Apoptosis was detected by TUNEL staining using the In Situ Cell Death Detection Kit, Fluorescein, following the manufacturer’s instructions. Positive cells were counted, and at least 10 fields per section for each sample were examined.

### Cell culture and treatment

Mouse proximal tubular epithelial cells (TKPTs), provided by Dr. Elsa Bella-Reuss (University of Texas Medical Branch, Galveston, TX, USA), were cultured in Dulbecco’s modified Eagle’s medium (DEME) with F12 containing 5% fetal bovine serum (FBS) and 0.5% penicillin and streptomycin in an atmosphere of 5% CO_2_ and 95% air at 37 °C. To investigate the effect of SET8 inhibition on TKPTs in response to cisplatin, cells were exposed to cisplatin (20 μM) for 24 h in the absence or presence of UNC0379 (10 μM) before being harvested for protein analysis.

### Transfection of siRNA into cells

Cells were seeded to 30–40% confluence in an antibiotic-free medium and then were transfected with siRNA specific to SET8, PTEN, and p53 according to the manufacturer’s instructions. Control siRNA (siCON) was used as a control for off-target changes in TKPTs. Twenty-four hours later, the medium was changed to a normal culture medium, and cells were treated for subsequent experiments.

### Transfection of PTEN and green fluorescent protein-tagged LC3B (GFP-LC3B) in TKPTs

PTEN plasmid (135676), Empty Vector plasmid (17448), and GFP-LC3B (21074), purchased from Addgene (Watertown, MA, USA), were transfected into TKPTs with Lipofectamine 3000 according to the manufacturer’s instructions. After being exposed to cisplatin for an additional 24 h, with or without UNC0379, cells were harvested and used for further analysis.

### Nuclear and cytoplasmic extraction

Cells were harvested and washed twice with cold PBS. Nuclear and cytoplasmic extraction was carried out using the NE-PER Nuclear and Cytoplasmic Extraction Reagents Kit (78833) that was purchased from Thermo Fisher Scientific (Waltham, MA, USA) according to the manufacturer’s instructions. Immunoblot analysis was used to assess the results of nuclear and cytoplasmic extraction by measuring the expression of histone H3 (a nuclear protein) and Tubulin (a cytoplasm protein).

### Cell viability assay

Cell viability was determined using a CCK-8 assay, according to the manufacturer’s protocol. Cells were seeded in 96-well plates and then with siRNA and/or pretreated with UNC0379 (10 μM) for 1 h, followed by exposure to cisplatin (20 μM). After treatment for 24 h, cells were exposed to CCK-8 solution for 1 h, and absorbance at 490 nm was measured using a microplate reader.

### RNA extraction, real-time PCR

The RNA Extraction Kit was obtained from Takara (Shiga, Japan). The extracted RNA underwent reverse transcription using a reverse transcription kit (RR036A, Takara, Japan). Real-time PCR was conducted with TB Green Premix EX TaqTM II (RR820A, Takara, Japan). The PCR primers are listed in Supplementary Table 3. PCR data were normalized relative to mouse β-Actin expression and analyzed using the 2^−ΔΔCt^ method.

### Western blotting analysis

Western blotting was performed as previously described [44]. The intensity of immunoblot results was determined by Image J software (v1.54d, NIH).

### Statistical analysis

Statistical analysis of the data was performed using GraphPad Prism version 9.0 (GraphPad Software, San Diego, CA, USA). Comparison between groups was evaluated using one-way ANOVA, followed by the student–Newman–Keuls test. *P* < 0.05 was considered statistically significant. All experiments were repeated three times or more and presented as mean ± SEM.

## Supplementary information

SET8 inhibition preserves PTEN to attenuate kidney cell apoptosis in cisplatin nephrotoxicity

Original data

## Data Availability

The published article includes all data sets generated/analyzed for this study.
