# Supplementary material for: SET8 inhibition preserves PTEN to attenuate kidney cell apoptosis in cisplatin nephrotoxicity
Source: Cell Death Dis. 2025 Mar 31;16(1):226. doi: 10.1038/s41419-025-07526-y (PMC11958763; doi:10.1038/s41419-025-07526-y)
Supplement: Supplementary file 1 — SET8 inhibition preserves PTEN to attenuate kidney cell apoptosis in cisplatin nephrotoxicity [file 41419_2025_7526_MOESM1_ESM.pdf]

Supplementary Table 1. Details of primary antibodies used for immunoblotting analysis

Antibody	Catalogue number	Supplier	Dilution
SET8	MA5-14804	Themo fisher Scientific (Waltham, MA, USA)	1:1000
cleaved-cas3	9664	Cell Signaling Technology (Danvers, MA, USA)	1:1000
p-p53(ser15)	9284	Cell Signaling Technology (Danvers, MA, USA)	1:1000
p53	2524	Cell Signaling Technology (Danvers, MA, USA)	1:1000
Bax	2772	Cell Signaling Technology (Danvers, MA, USA)	1:1000
Bcl-X_L	2764	Cell Signaling Technology (Danvers, MA, USA)	1:1000
E-cadherin	14472	Cell Signaling Technology (Danvers, MA, USA)	1:1000
PTEN	9559	Cell Signaling Technology (Danvers, MA, USA)	1:1000
p-ERK	9101	Cell Signaling Technology (Danvers, MA, USA)	1:1000
ERK	4695	Cell Signaling Technology (Danvers, MA, USA)	1:1000
p-p38	4511	Cell Signaling Technology (Danvers, MA, USA)	1:1000
p38	9212	Cell Signaling Technology (Danvers, MA, USA)	1:1000
FOXO1	28807	Cell Signaling Technology (Danvers, MA, USA)	1:1000
p-H2A	9718	Cell Signaling Technology (Danvers, MA, USA)	1:1000
H2A	7631	Cell Signaling Technology (Danvers, MA, USA)	1:1000
p21	Ab107099	Abcam (Cambridge, MA, USA)	1:1000
NGAL	AF857	R&D systems (Minneapolis, MN, USA)	1:1000
Kim-1	AF1817	R&D systems (Minneapolis, MN, USA)	1:1000
CHMP2A	10477-1-AP	ProteinTech (Rosemont, IL, USA)	1:1000
H4K20me1	sc-134221	Santa Cruz Biotechnology (Dallas, TX, USA)	1:500
Tubulin	sc-5286	Santa Cruz Biotechnology (Dallas, TX, USA)	1:1000
β-actin	sc-47778	Santa Cruz Biotechnology (Dallas, TX, USA)	1:1000
GAPDH	sc-32233	Santa Cruz Biotechnology (Dallas, TX, USA)	1:1000
Histone H4	07-108	Sigma-Aldrich (St. Louis, MO, USA)	1:1000

Supplementary Table 2. Details of primary antibodies used for immunofluorescence staining.

Antibody	Catalogue number	Supplier	Dilution
SET8	MA5-14804	Themo fisher Scientific (Waltham, MA, USA)	1:200
NGAL	AF857	R&D systems (Minneapolis, MN, USA)	1:200
PTEN	60300-1-Ig	ProteinTech (Rosemont, IL, USA)	1:200

Supplementary table 3. Primers used for Real-Time PCR

Gene	Type	Sequence (5’-3’)
PTEN	Forward Primer	TGGATTCGACTTAGACTTGACCT
	Reverse Primer	GCGGTGTCATAATGTCTCTCAG
SET8	Forward Primer	CAGACCAAACTGCACGACATC
	Reverse Primer	CTTGCTTCGGTCCCCATAGT
β-Actin	Forward Primer	GGCTGTATTCCCCTCCATCG
	Reverse Primer	CCAGTTGGTAACAATGCCATGT

Figure S1

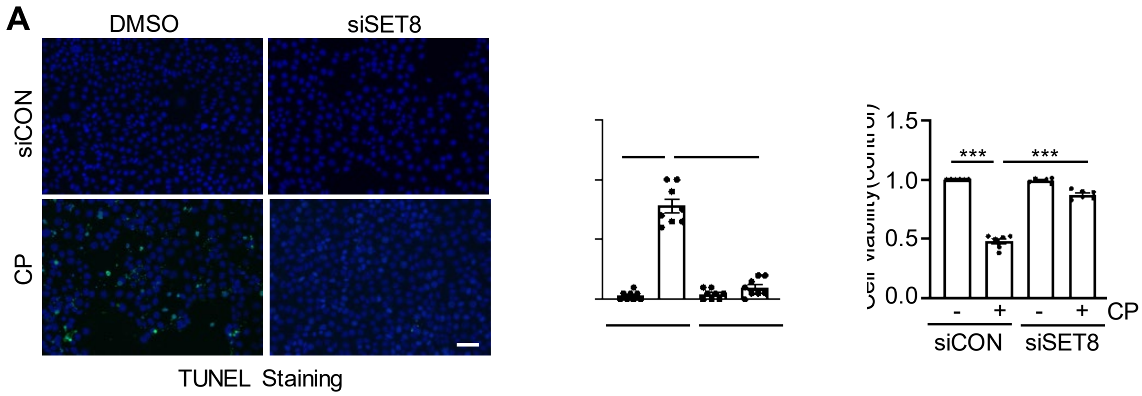

Supplementary Figure 1. Silencing of SET8 attenuates apoptosis in cultured TKPTs exposed to cisplatin (CP). Cultured TKPTs were transfected with negative control siRNA (siCON) or specific siRNA for SET8 (siSET8) and then exposed to CP for another 24 hours. (A) TUNEL staining was conducted 24 hours after administering CP, and TUNEL-positive cells were calculated by at least 10 fields per section. Scale bar = 50 μm. (C) Cell viability was detected 24 hours later by cell counting kit 8 (CCK8). Data are represented as the mean± SEM of at least three experiments. ***P<0.001.

Figure S2

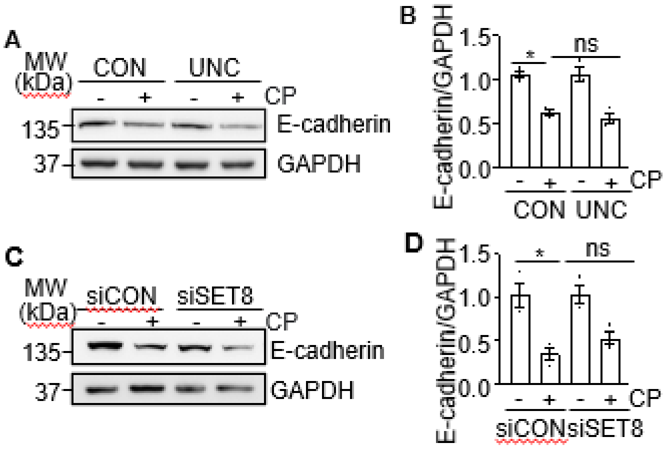

Supplementary Figure 2. Inhibition of SET8 does not affect cisplatin (CP)-induced downregulation of E-cadherin in cultured TKPTs. TKPTs were pretreated with UNC0379 for 1 hour (A) or transfected with siRNA targeting SET8 (siSET8) or control siRNA (siCON) (C) for 24 hours and then exposed to CP for 24 hours. The expression level of E-cadherin was quantified by densitometry and normalized by GAPDH (B, D). Data are represented as the mean± SEM of at least three experiments. *P < 0.05.

Figure S3

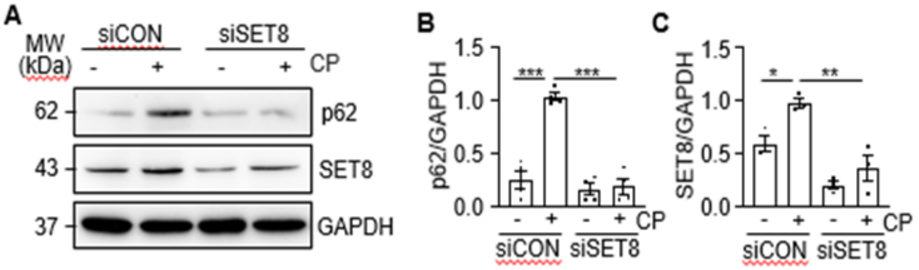

Supplementary Figure 3. Inhibition of SET8 inhibits CP-induced expression of p62 in cultured TKPTs. TKPTs were transfected with siRNA targeting SET8 (siPTEN) or control siRNA (siCON) for 24 hours and then exposed to cisplatin for 24 hours. Cell lysates were subject to immunoblot analysis using antibodies against p62 or SET8 (A). The expression levels of p62 (B) and SET8 (C) were quantified by densitometry and normalized by GAPDH. Data are represented as the mean± SEM of at least three experiments. *P < 0.05, **P < 0.01, ***P<0.001.

Figure S4

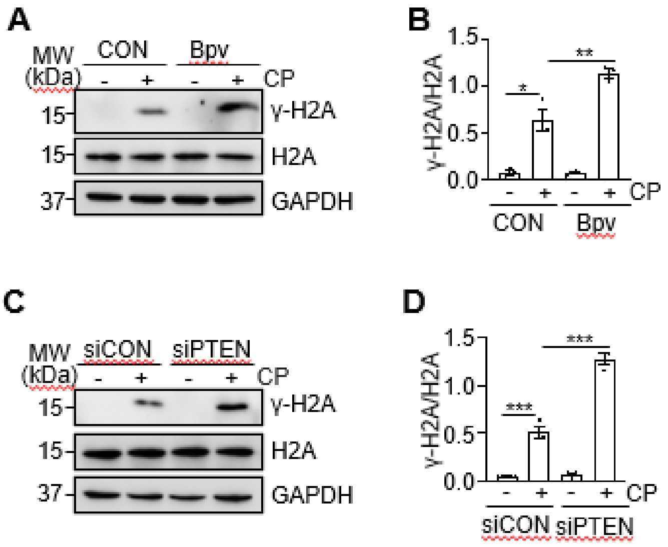

Supplementary Figure 4. Inhibition of PTEN aggravated cisplatin-induced DNA damage in cultured TKPTs. TKPTs were pretreated with Bpv for 1 hour (A) or transfected with siRNA targeting PTEN (siPTEN) or control siRNA (siCON) (C) for 24 hours and then exposed to cisplatin for 24 hours. The expression level of γ-H2A was quantified by densitometry and normalized by H2A (B, D). Data are represented as the mean± SEM of at least three experiments. *$P < 0.05$, **$P < 0.01$, ***$P<0.001$.

Figure S5

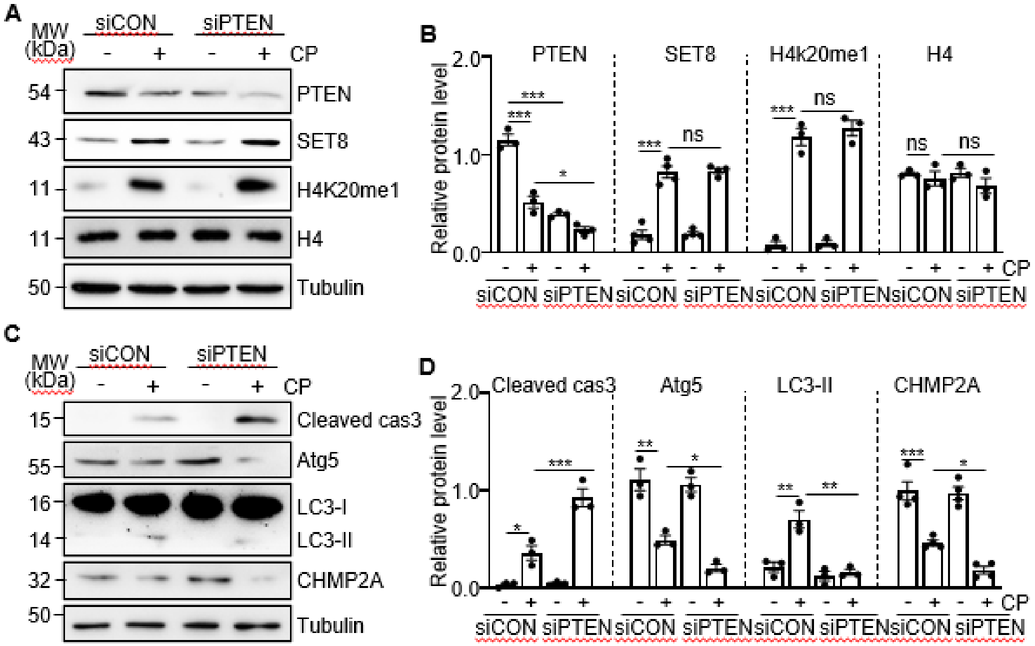

Supplementary Figure 5. Inhibition of PTEN potentiates cisplatin (CP)-induced impairment of autophagy. TKPTs were treated with CP for 24 hours after transfection of siRNA targeting PTEN (siPTEN) or control siRNA (siCON) for 24 hours (A-D). Cell lysates were prepared and subjected to immunoblot analysis using antibodies as indicated (A, E). The levels of all the proteins were quantified by densitometry, the expression of PTEN, SET8, H4, cleaved cas-3, Atg5, CHMP2A was normalized with tubulin, and the expression of LC3-II was normalized with LC3-I, respectively (B, D). Data are represented as the mean± SEM of at least three experiments. *P < 0.05, **P < 0.01, ***P<0.001.

Figure S6

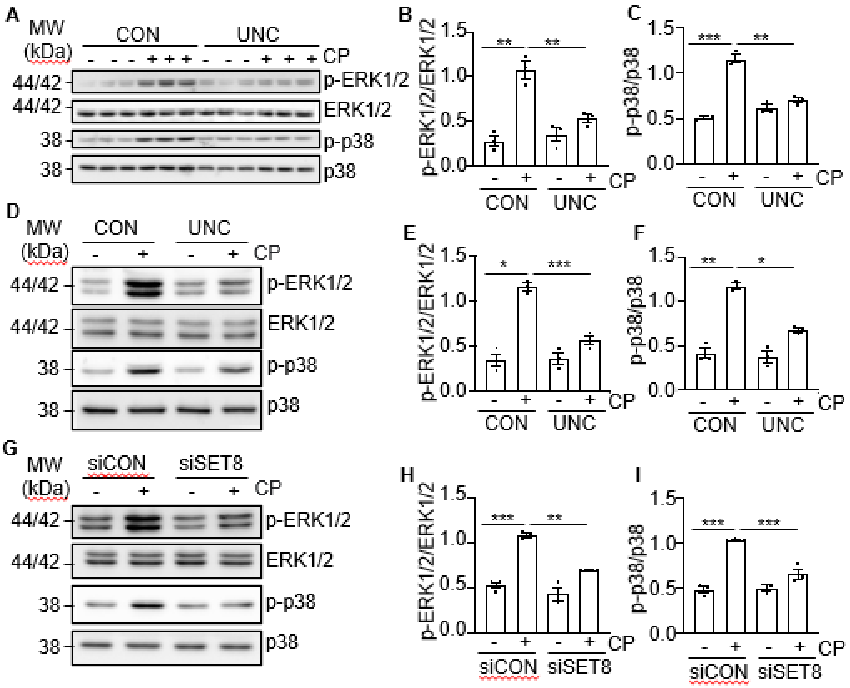

Supplementary Figure 6. Inhibition of SET8 decreased cisplatin (CP)-induced upregulation of extracellular signal-regulated kinase 1/2 (ERK1/2) and p38 pathways in mice and cultured TKPTs. (A, D, G) Kidney tissue lysates were subject to immunoblot analysis using antibodies against p-ERK1/2, ERK1/2, p-p38, and p38. The levels of p-ERK1/2 and p-p38 were quantified by densitometry and normalized with ERK1/2 and p38 as indicated (B-C, E-F, H-I). Data are represented as the mean ± SEM of at least three experiments. *P < 0.05, **P < 0.01, ***P<0.001.
