# Supplementary material for: SET8 inhibition preserves PTEN to attenuate kidney cell apoptosis in cisplatin nephrotoxicity
Source: Cell Death Dis. 2025 Mar 31;16(1):226. doi: 10.1038/s41419-025-07526-y (PMC11958763; doi:10.1038/s41419-025-07526-y)
Supplement: Supplementary file 2 — Original data [file 41419_2025_7526_MOESM2_ESM.pptx]

## Slide 1
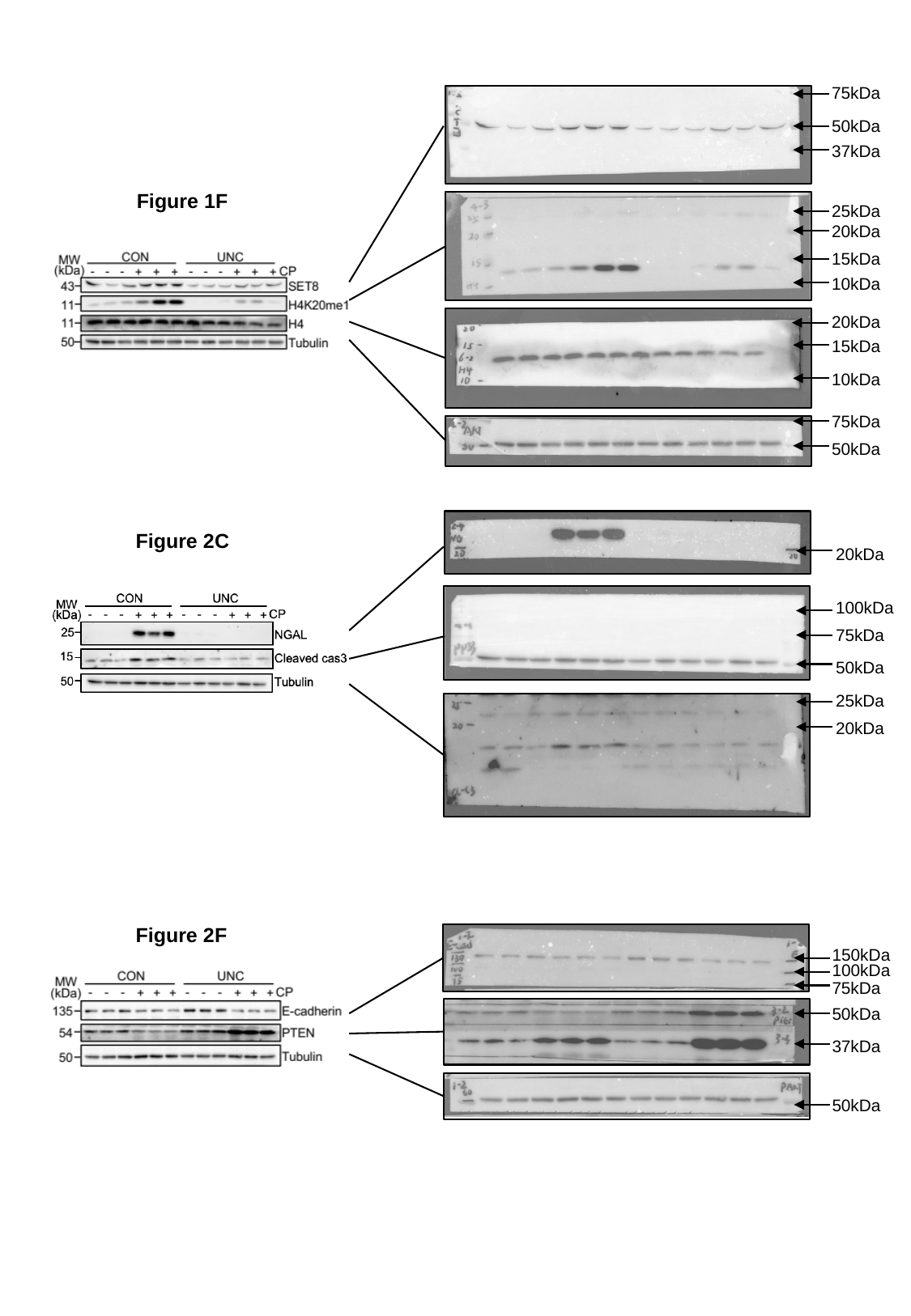

75kDa
50kDa
37kDa
Figure 1F
25kDa
20kDa
15kDa
10kDa
20kDa
15kDa
10kDa
75kDa
50kDa
Figure 2C
20kDa
100kDa
75kDa
50kDa
25kDa
20kDa
Figure 2F
150kDa
100kDa
75kDa
50kDa
37kDa
50kDa

## Slide 2
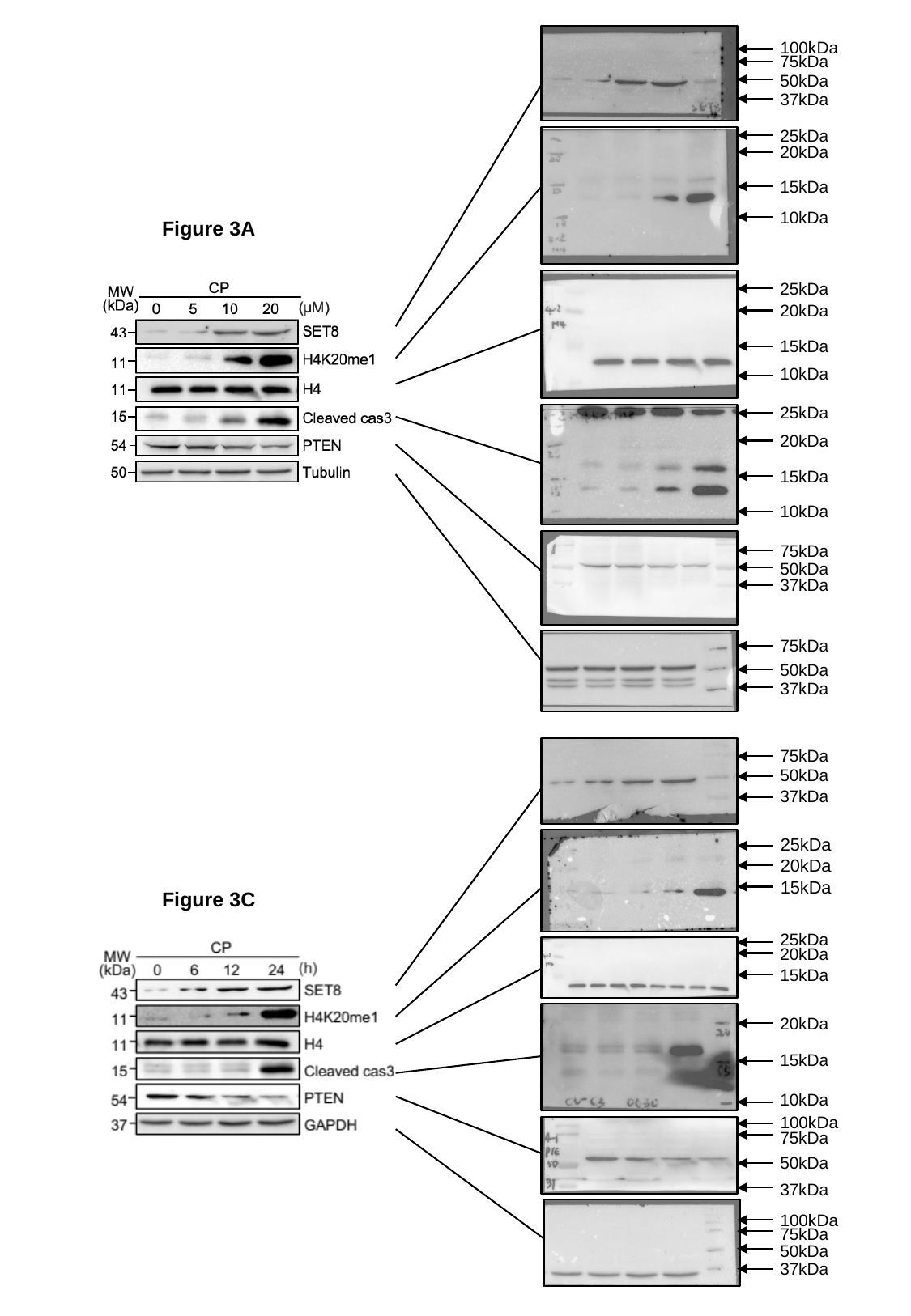

100kDa
75kDa
50kDa
37kDa
25kDa
20kDa
15kDa
10kDa
Figure 3A
25kDa
20kDa
15kDa
10kDa
25kDa
20kDa
15kDa
10kDa
75kDa
50kDa
37kDa
75kDa
50kDa
37kDa
75kDa
50kDa
37kDa
25kDa
20kDa
15kDa
Figure 3C
25kDa
20kDa
15kDa
20kDa
15kDa
10kDa
100kDa
75kDa
50kDa
37kDa
100kDa
75kDa
50kDa
37kDa

## Slide 3
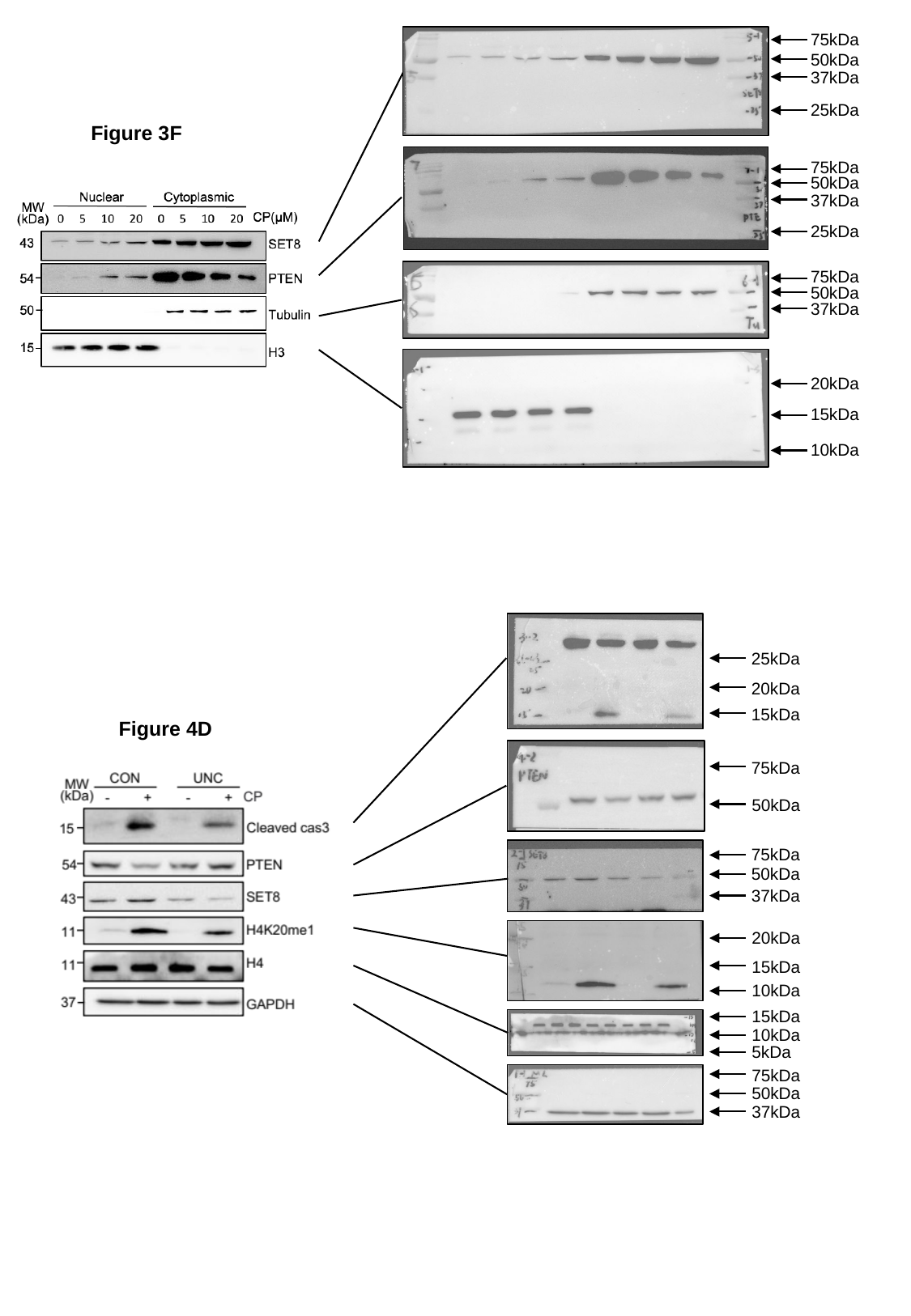

75kDa
50kDa
37kDa
25kDa
Figure 3F
75kDa
50kDa
37kDa
25kDa
75kDa
50kDa
37kDa
20kDa
15kDa
10kDa
25kDa
20kDa
15kDa
Figure 4D
75kDa
50kDa
75kDa
50kDa
37kDa
20kDa
15kDa
10kDa
15kDa
10kDa
5kDa
75kDa
50kDa
37kDa

## Slide 4
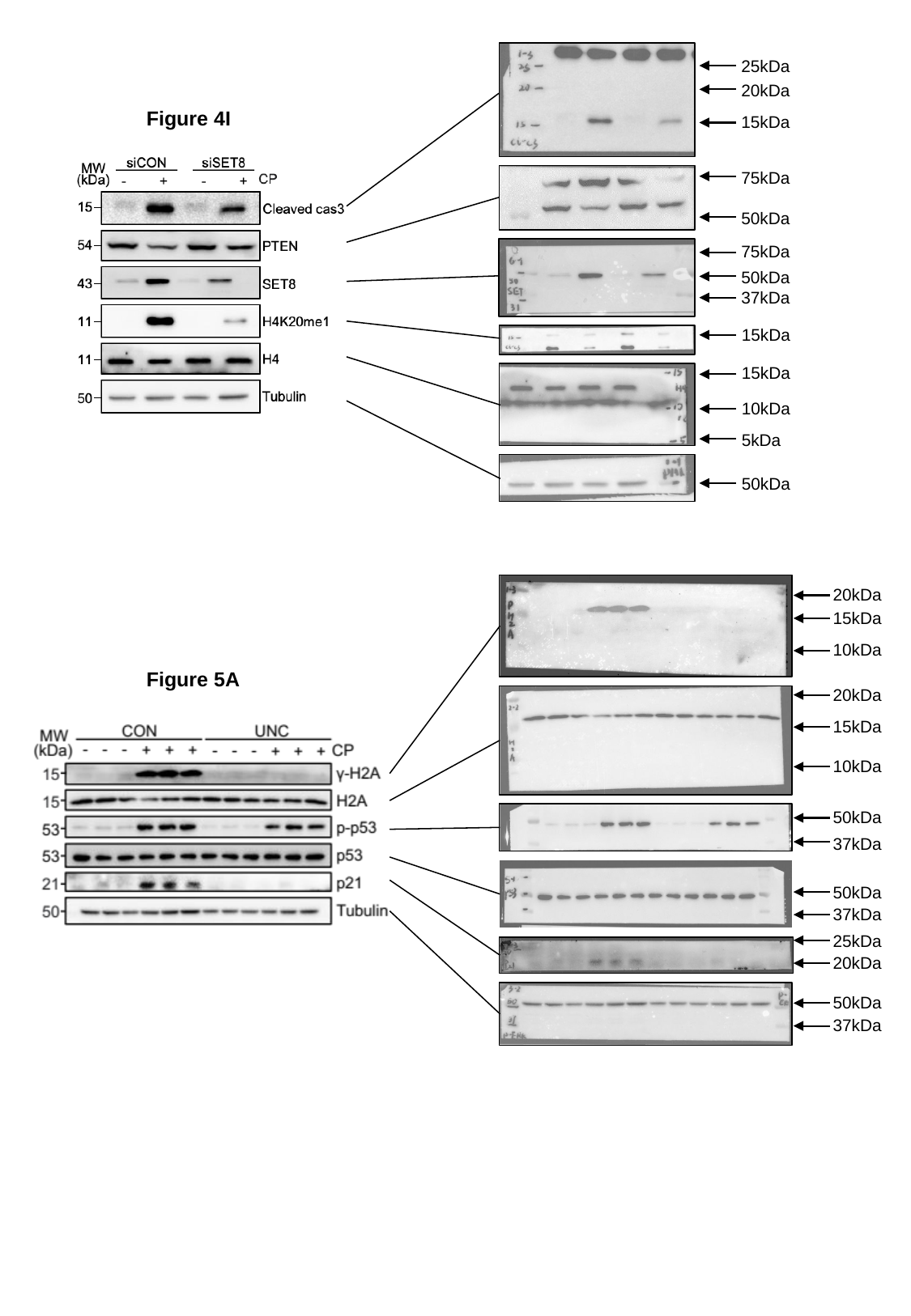

25kDa
20kDa
Figure 4I
15kDa
75kDa
50kDa
75kDa
50kDa
37kDa
15kDa
15kDa
10kDa
5kDa
50kDa
20kDa
15kDa
10kDa
Figure 5A
20kDa
15kDa
10kDa
50kDa
37kDa
50kDa
37kDa
25kDa
20kDa
50kDa
37kDa

## Slide 5
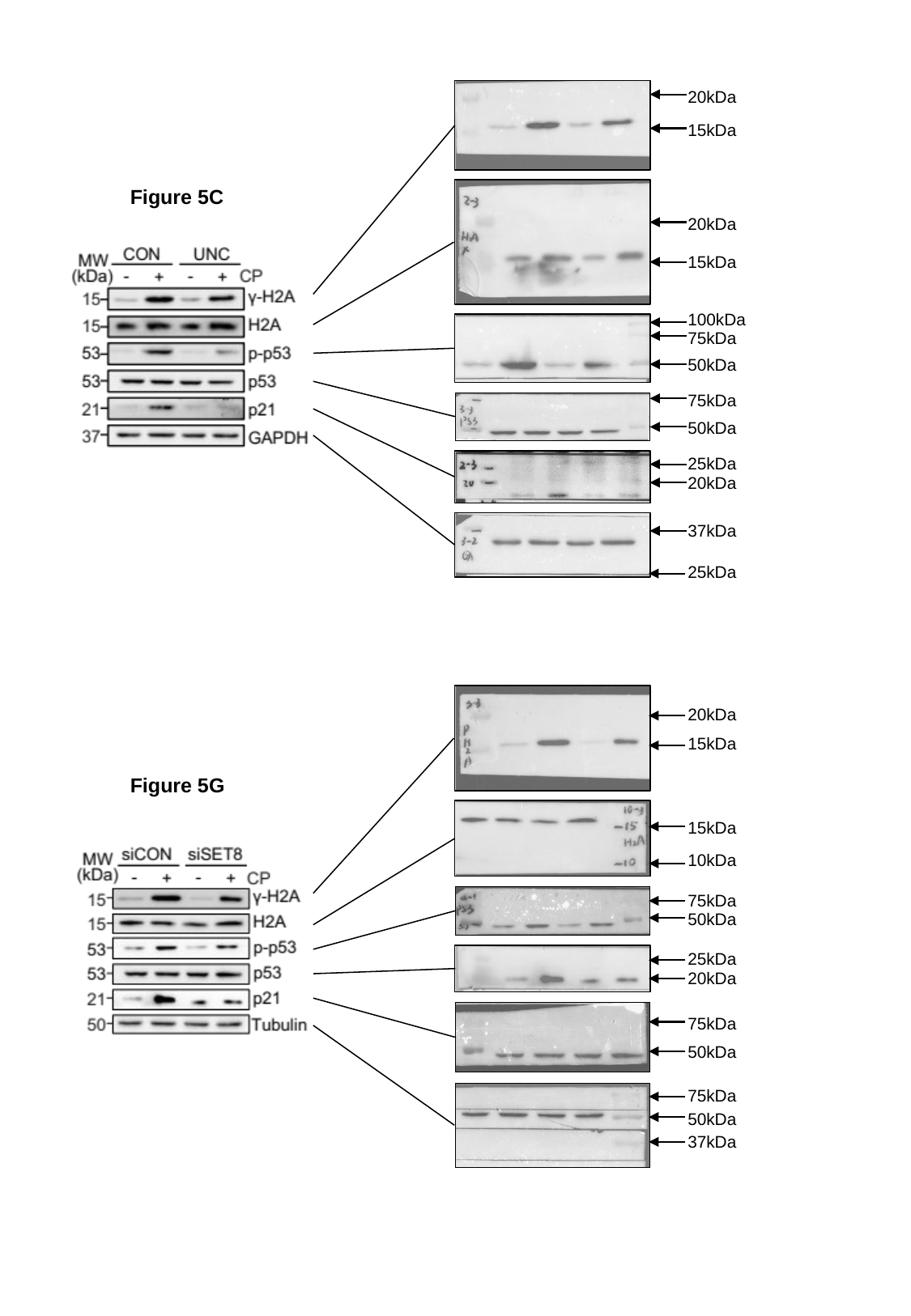

20kDa
15kDa
Figure 5C
20kDa
15kDa
100kDa
75kDa
50kDa
75kDa
50kDa
25kDa
20kDa
37kDa
25kDa
20kDa
15kDa
Figure 5G
15kDa
10kDa
75kDa
50kDa
25kDa
20kDa
75kDa
50kDa
75kDa
50kDa
37kDa

## Slide 6
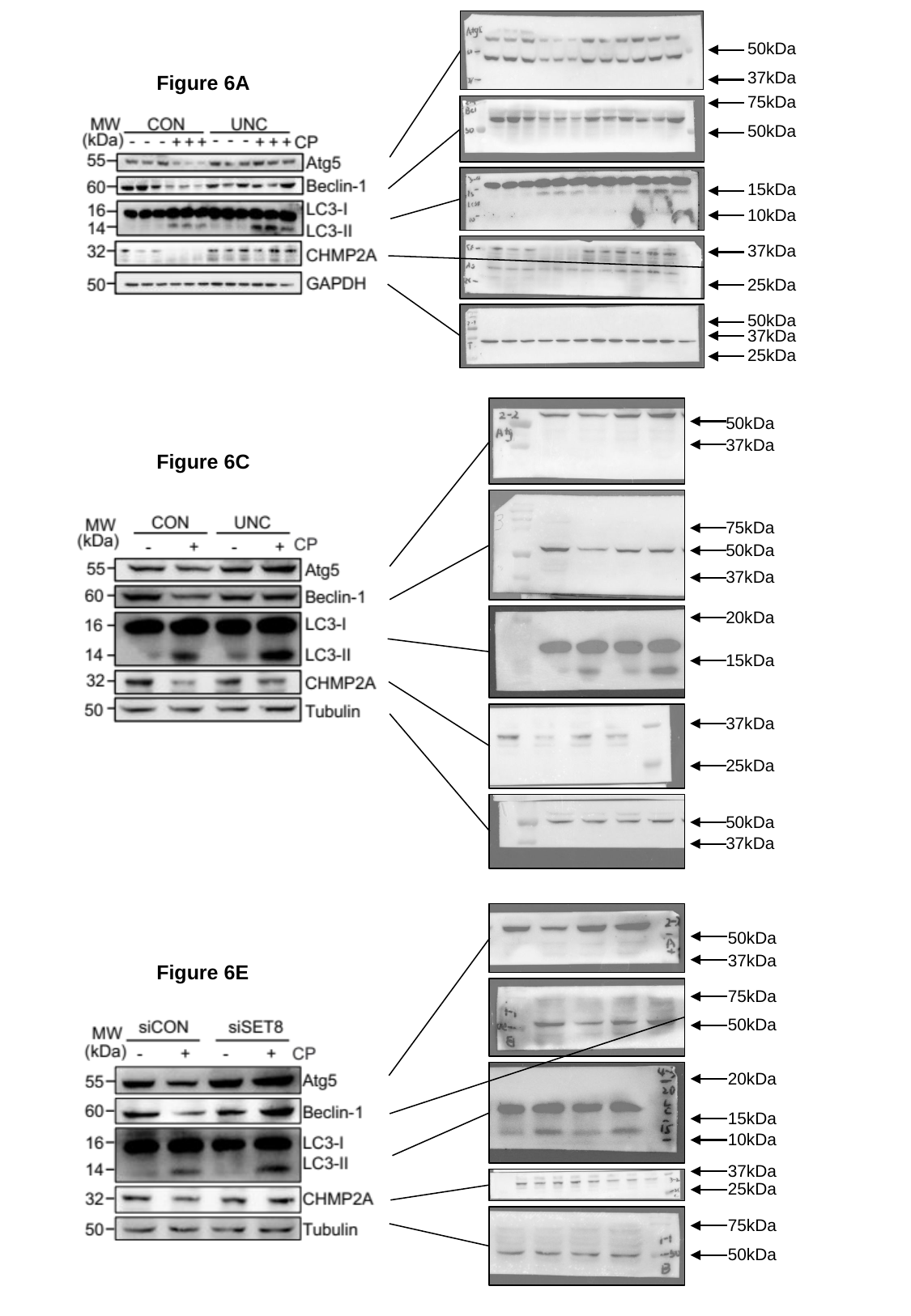

50kDa
37kDa
Figure 6A
75kDa
50kDa
15kDa
10kDa
37kDa
25kDa
50kDa
37kDa
25kDa
50kDa
37kDa
Figure 6C
75kDa
50kDa
37kDa
20kDa
15kDa
37kDa
25kDa
50kDa
37kDa
50kDa
37kDa
Figure 6E
75kDa
50kDa
20kDa
15kDa
10kDa
37kDa
25kDa
75kDa
50kDa

## Slide 7
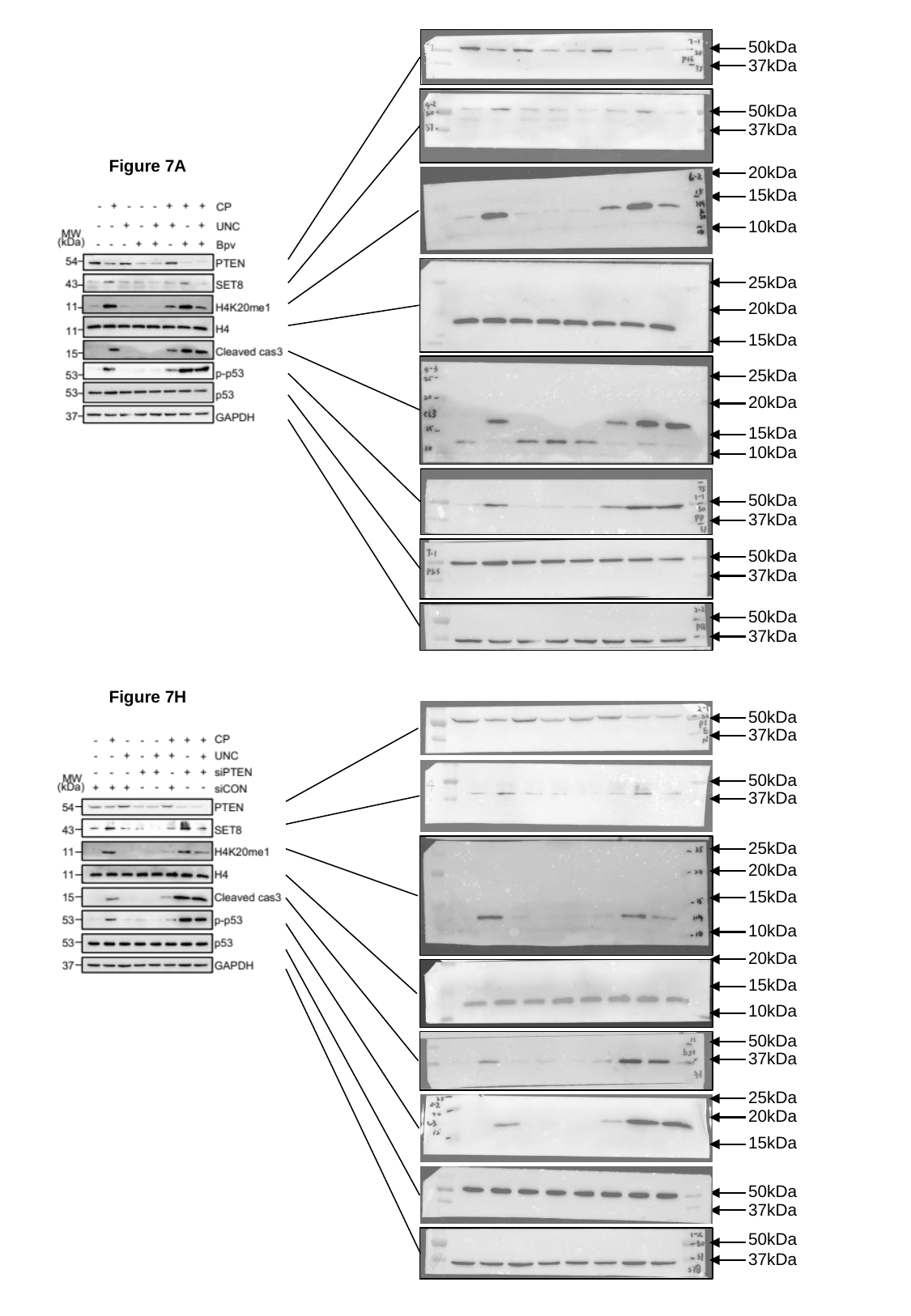

50kDa
37kDa
50kDa
37kDa
Figure 7A
20kDa
15kDa
10kDa
25kDa
20kDa
15kDa
25kDa
20kDa
15kDa
10kDa
50kDa
37kDa
50kDa
37kDa
50kDa
37kDa
Figure 7H
50kDa
37kDa
50kDa
37kDa
25kDa
20kDa
15kDa
10kDa
20kDa
15kDa
10kDa
50kDa
37kDa
25kDa
20kDa
15kDa
50kDa
37kDa
50kDa
37kDa

## Slide 8
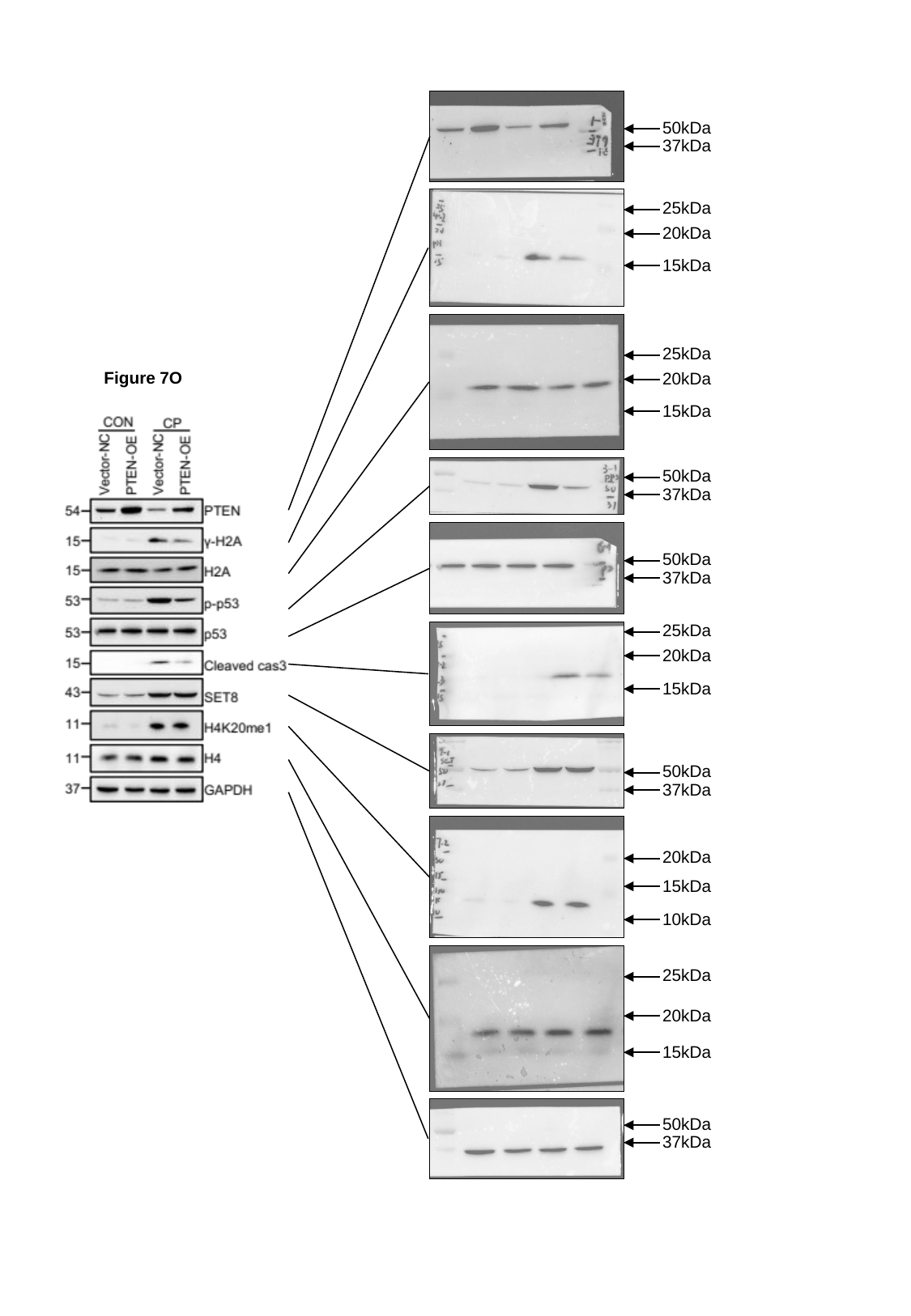

50kDa
37kDa
25kDa
20kDa
15kDa
25kDa
Figure 7O
20kDa
15kDa
50kDa
37kDa
50kDa
37kDa
25kDa
20kDa
15kDa
50kDa
37kDa
20kDa
15kDa
10kDa
25kDa
20kDa
15kDa
50kDa
37kDa

## Slide 9
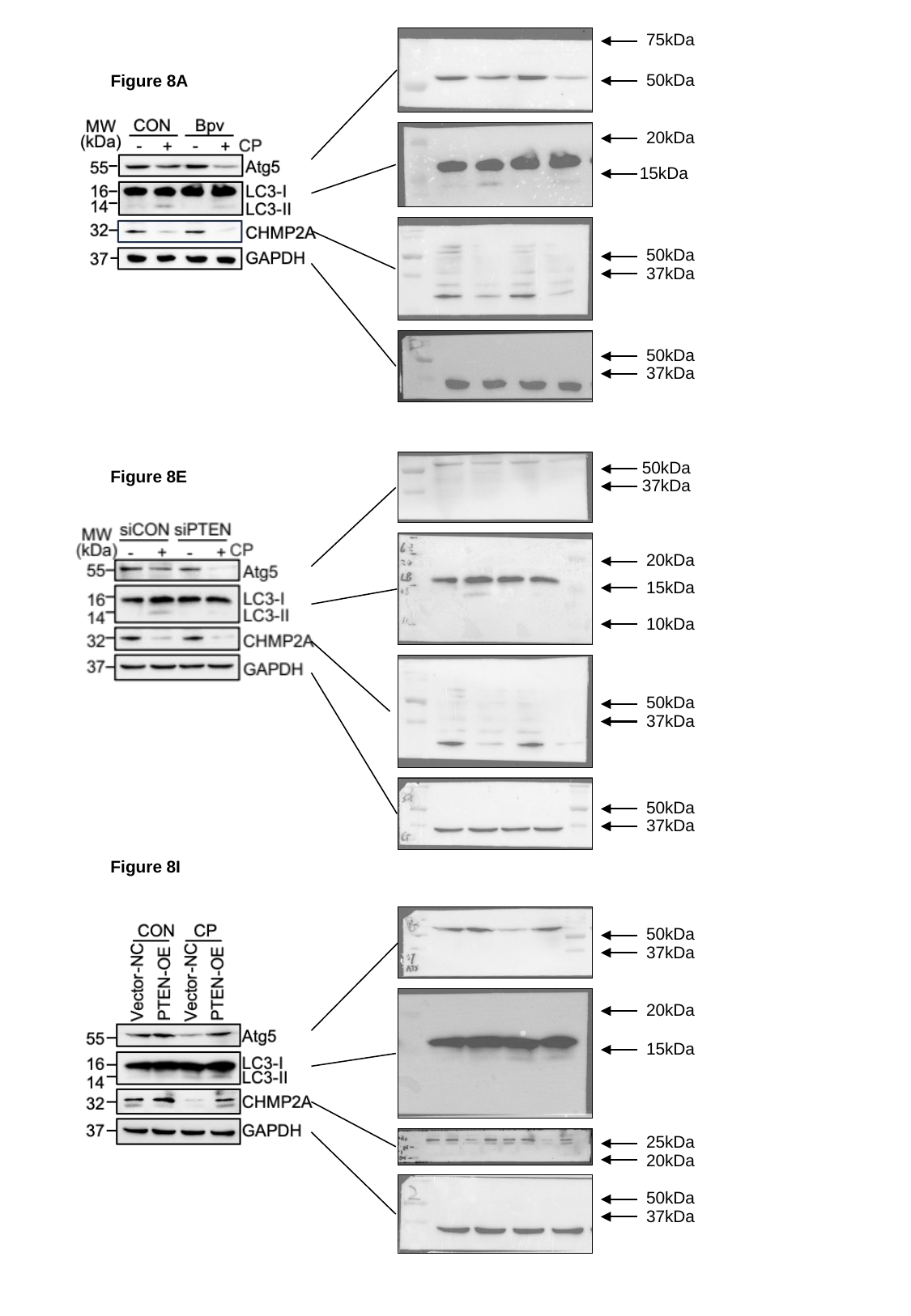

75kDa
50kDa
Figure 8A
20kDa
15kDa
50kDa
37kDa
50kDa
37kDa
50kDa
Figure 8E
37kDa
20kDa
15kDa
10kDa
50kDa
37kDa
50kDa
37kDa
Figure 8I
50kDa
37kDa
20kDa
15kDa
25kDa
20kDa
50kDa
37kDa

## Slide 10
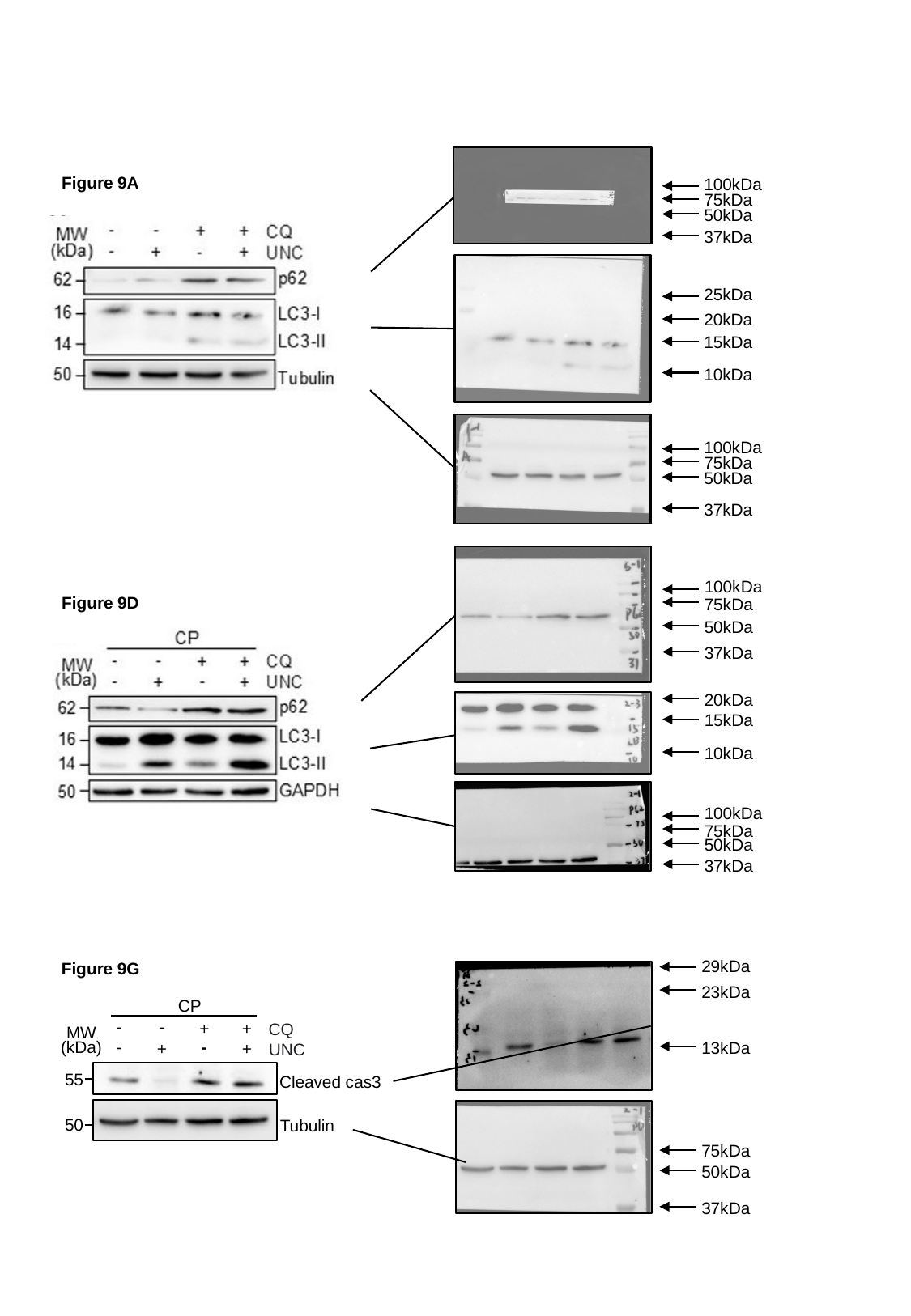

Figure 9A
100kDa
75kDa
50kDa
37kDa
25kDa
20kDa
15kDa
10kDa
100kDa
75kDa
50kDa
37kDa
100kDa
Figure 9D
75kDa
50kDa
37kDa
20kDa
15kDa
10kDa
100kDa
75kDa
50kDa
37kDa
29kDa
Figure 9G
23kDa
CP
-
-
+
+
CQ
 MW
 (kDa)
-
-
13kDa
+
+
UNC
55
Cleaved cas3
50
Tubulin
75kDa
50kDa
37kDa

## Slide 11
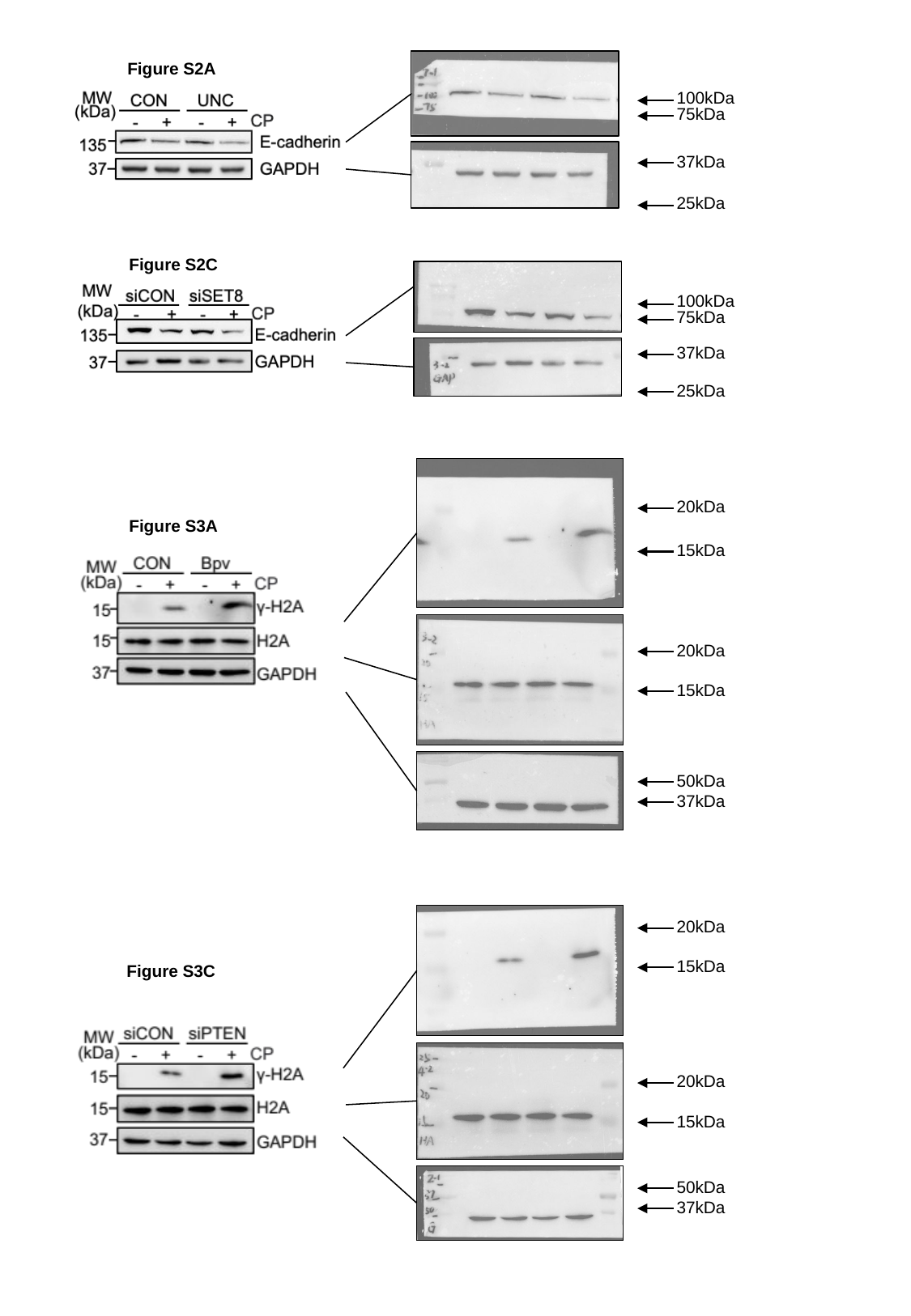

Figure S2A
100kDa
75kDa
37kDa
25kDa
Figure S2C
100kDa
75kDa
37kDa
25kDa
20kDa
Figure S3A
15kDa
20kDa
15kDa
50kDa
37kDa
20kDa
15kDa
Figure S3C
20kDa
15kDa
50kDa
37kDa

## Slide 12
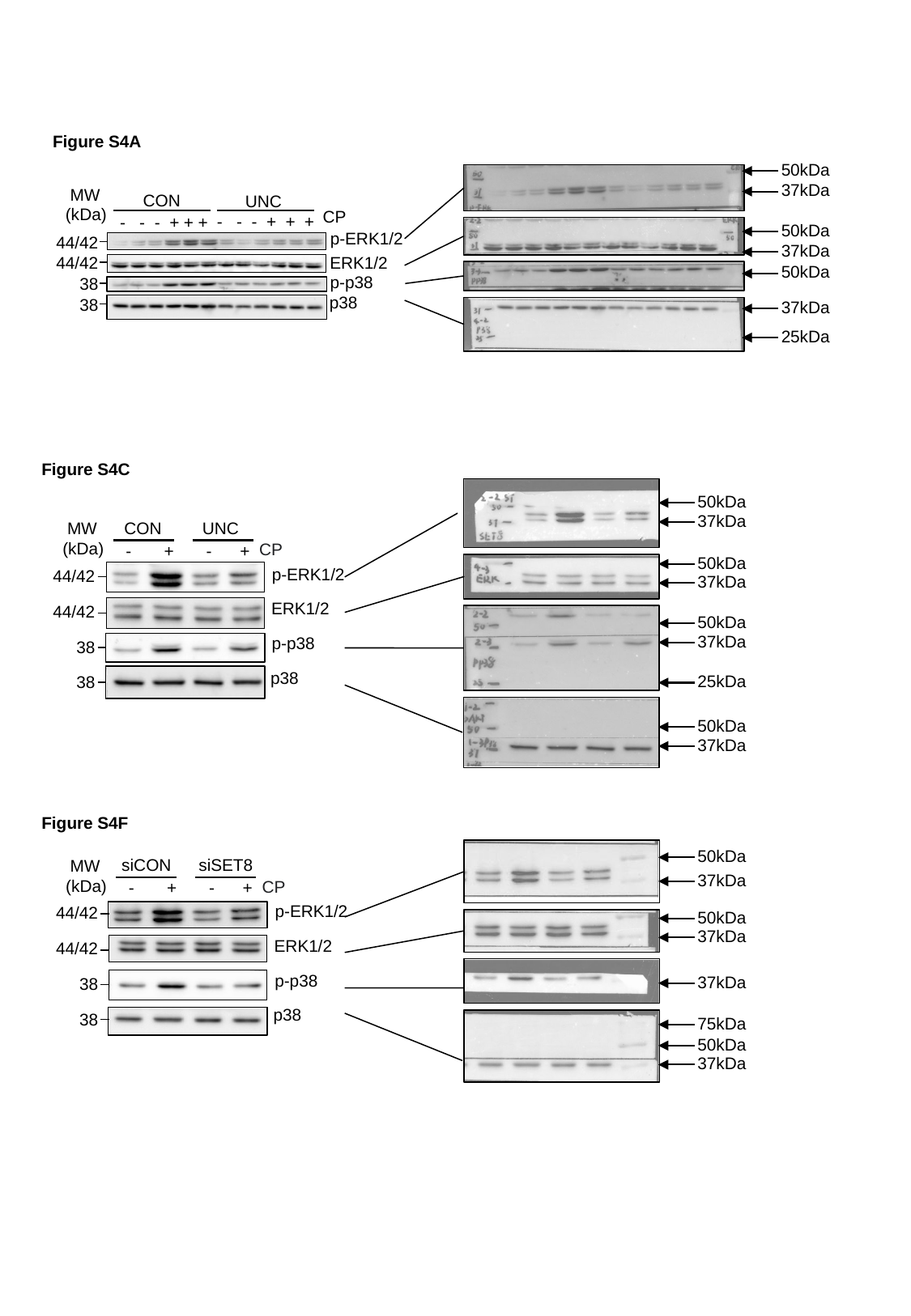

Figure S4A
50kDa
37kDa
 MW
(kDa)
CON
UNC
CP
 - - - + + +
 - - - + + +
50kDa
p-ERK1/2
44/42
37kDa
ERK1/2
44/42
50kDa
p-p38
38
p38
38
37kDa
25kDa
Figure S4C
50kDa
37kDa
CON
 MW
(kDa)
CP
 - + - +
UNC
p-ERK1/2
44/42
ERK1/2
44/42
p-p38
38
p38
38
50kDa
37kDa
50kDa
37kDa
25kDa
50kDa
37kDa
Figure S4F
50kDa
siCON
 MW
(kDa)
CP
 - + - +
siSET8
p-ERK1/2
44/42
ERK1/2
44/42
p-p38
38
p38
38
37kDa
50kDa
37kDa
37kDa
75kDa
50kDa
37kDa
